# Feature Detection of Non-Cooperative and Rotating Space Objects through Bayesian Optimization

**DOI:** 10.3390/s24154831

**Published:** 2024-07-25

**Authors:** Rabiul Hasan Kabir, Xiaoli Bai

**Affiliations:** Department of Mechanical and Aerospace Engineering, Rutgers University, New Brunswick, NJ 08901, USA; rabiulhasan.kabir@rutgers.edu

**Keywords:** Bayesian Optimization, Gaussian Process, proximity operation, non-cooperative rotating space object, active SLAM, space-domain awareness

## Abstract

In this paper, we propose a Bayesian Optimization (BO)-based strategy using the Gaussian Process (GP) for feature detection of a known but non-cooperative space object by a chaser with a monocular camera and a single-beam LIDAR in a close-proximity operation. Specifically, the objective of the proposed Space Object Chaser-Resident Assessment Feature Tracking (SOCRAFT) algorithm is to determine the camera directional angles so that the maximum number of features within the camera range is detected while the chaser moves in a predefined orbit around the target. For the chaser-object spatial incentive, rewards are assigned to the chaser states from a combined model with two components: feature detection score and sinusoidal reward. To calculate the sinusoidal reward, estimated feature locations are required, which are predicted by Gaussian Process models. Another Gaussian Process model provides the reward distribution, which is then used by the Bayesian Optimization to determine the camera directional angles. Simulations are conducted in both 2D and 3D domains. The results demonstrate that SOCRAFT can generally detect the maximum number of features within the limited camera range and field of view.

## 1. Introduction

The rapid increase in human-made space debris in Low Earth Orbit (LEO) poses a serious threat to current and future space missions. With the increasing number of artificial objects from satellite operations, there is an increase in space debris [1]. Space debris consists of varying sizes of space objects, starting from decommissioned satellites and discarded rocket engines to paint chips and small remnants from collisions among space objects. These uncontrolled space objects cause a severe threat since even the small ones can initiate a chain collision among space debris, known as the Kessler syndrome [2]. To guarantee the safety of ongoing and future space missions, NASA and other space agencies around the world are planning to initiate space object removal projects using space robotics (e.g., in-orbit service, manufacturing, and assembly—ISAM [3]), which require direct interactions between a controllable spacecraft, also known as a chaser, and a non-cooperative space object (e.g., decommissioned satellites).

It is relatively straightforward to plan how to interact and remove a space object with known dynamics, geometry, and physical parameters (e.g., mass, mass moment of inertia, center of mass, etc.). However, due to collisions among space objects and atmospheric drag, a space object’s geometry may vary, and its trajectory can change. These changes make it difficult to exactly know the space object’s geometry, position, and trajectory with certainty. Interacting directly with such space objects, especially those that are uncontrollable, can lead to catastrophic events. Furthermore, a non-cooperative space object cannot relay any information regarding its state or dynamics, adding more complexity to the task of space debris removal. Therefore, gathering knowledge about a non-cooperative space object’s current geometry, physical parameters, and dynamics through observations is crucial to space debris removal operations.

A critical step in gathering the necessary information regarding an unknown and non-cooperative space object is to create a 3D model of the object  [4], which is essential for planning how to safely and efficiently execute the space object removal task. This objective requires mapping the object with a sensor such as a camera or a LIDAR and then utilizing computer vision algorithms to convert the collected information into a 3D target map. In the presence of noise associated with the chaser state, this mapping task can be considered as a Simultaneous Mapping and Localization (SLAM) problem.

The SLAM problem, which is primarily linked to the field of robotics, has been investigated by researchers over the last few decades to map an unknown environment with the help of a moving and sensing robotic agent under uncertainty and at the same time, to utilize the gathered environment information to improve the certainty of the state of the agent. Due to SLAM’s relevancy in a wide range of applications, such as underwater [5], underground mines [6], ground [7] and aerial [8] mapping, researchers have come up with different solutions for the SLAM problem, for example, Extended Kalman Filter (EKF)-SLAM [9], FastSLAM [10], iSAM [11], occupancy grid method [12], etc.

Space applications, specifically, the mapping of space objects, require dealing with dynamic environments or space objects. Tweddle investigated creating a 3D model and determining the state and some physical parameters of an unknown, non-cooperative, and spinning target [13]. In this study, an inertially stationary chaser spacecraft with an onboard stereo camera system is considered as an observer and tasked with capturing images of the target object. The captured stereo images are then processed through Random Sample Consensus (RANSAC) [14] and Scale Invariant Feature Transform (SIFT) [15] to extract useful information regarding feature detection and matching of the target object. The feature information is then utilized to solve a SLAM problem using a modified version of the iSAM [11] algorithm, to create a 3D map and determine various components of the state of the target. One limitation of this work is the assumption of an inertially stationary observer. In Ref.  [16], Setterfield addressed this limitation and considered dynamic objects. The main contributions of Setterfield’s work in Ref. [16] are to determine the trajectory of a moving observer using the factor graph approach, create the 3D model, and estimate the inertial properties of an unknown, non-cooperative, and spinning target.

The SLAM algorithms do not consider path planning and only apply to situations where the path to take is available before deployment. To handle path planning problems while performing SLAM, researchers have appended the planning component to the mapping and localization algorithms, which are called the Active SLAM or ASLAM. Relevant to space applications, an information theory-based algorithm is designed in Ref. [17], where the authors solved the relative localization problem between a stationary target satellite and a chaser spacecraft with an onboard visual camera and determined the optimal camera directional angle for collecting information regarding the target. That work assumed that the chaser circumnavigated the target in a fixed, planar, and circular orbit, and the target consisted of four stationary feature points tracked by the onboard camera. In contrast, in our paper, we consider rotating features and circular relative chaser orbits in both the 2D and 3D domains.

A more recent approach developed for on-orbit inspection missions is the Sampling-Based Model Predictive Optimization (SBMPO) algorithm. In Ref. [18], SBMPO is utilized to design a mission for a mapping spacecraft to observe and map the entire surface of the asteroid Didymain. In Ref. [19], the SBMPO is used for designing a passively safe on-orbit inspection mission for a chaser spacecraft to observe a known, non-cooperative tumbling target. To do so, candidate trajectories are assigned some scores and then, the best trajectory is obtained using a simple heuristic search method. The limitation of considering a known tumbling target is removed in Refs. [20,21], where the authors proposed a modified SBMPO algorithm to design trajectories for a chaser to inspect an unknown, non-cooperative tumbling target. The modified SBMPO is similar to Ref. [19] except the trajectory score includes a term for exploring the unknown target. Also, instead of using a simple heuristic search method to obtain the best trajectories, a more robust approach called the subset simulation (SS) is implemented.

One elementary component of designing any planning technique is the state estimation strategy to make predictions under state or environmental uncertainties. Past research related to space applications conducted for planning under uncertainty shows that physics-based methods such as the factor graph, EKF [22,23], or UKF [23] are the preferred methods for state estimation. However, it is well known that if the underlying physics model is not properly identified and modeled or the filter is not well tuned, physics-based methods cannot provide reliable state estimates. In such scenarios, data-driven models offer a more straightforward and accessible way for state estimation.

Recent improvements in computing capacity and Machine Learning (ML) algorithms have paved the way for implementing ML methodologies in Active SLAM problems for robotics [24,25] and space applications. In a contemporary work, a Reinforcement Learning (RL)-based path-planning method is proposed for a chaser spacecraft to create a 3D model of a non-cooperative target satellite [26]. Additionally, in a series of investigations, a Deep RL (DRL) algorithm called REINFORCE is utilized for an imaging spacecraft around an unknown small body in space [27,28]. These RL-based algorithms focus on reducing dependency on human effort for space-mission planning and autonomously developing optimal path-planning strategies from data-driven models. However, besides several undesirable assumptions made in these papers, these works indicate that RL-based algorithms require a lot of training time and data to design trajectories, which might limit their applications for practical scenarios.

Motivated by Ref. [17], we are interested in a scenario with the following assumptions. First, we assume that the target consists of four specific features. In general, features can either be regions [29], curves or lines [30], or points [31], which have higher visibility than their surrounding areas and thus are easily distinguishable in the captured images. In the context of the considered satellite proximity operations, the features indicate salient points located on the surface of the target such as corners of solar panels, endpoints of the poles on antennas, etc. They represent locations with significance for various purposes, for instance, grasping points for robotic capture. Second, the structure of the target is roughly known from prior operations. This assumption allows us to choose predefined natural closed relative orbits for the chaser close to the target without worrying about collisions. Due to this assumption, the chaser cannot change its trajectories, and the sensor onboard the chaser can only observe the target partially due to its limited range and field of view (FOV). Therefore, the only control variables are the sensor orientation angles, and as a result, the maximum number of feature detection considering the camera range and FOV is set as the mission objective. This scenario arises when the chaser needs to closely inspect the target to collect data in high resolution. Third, we assume that the chaser carries a monocular camera as well as a single-beam LIDAR sensor to collect information regarding the feature positions, similar to Ref. [32]. Unlike many previous works [33,34], we assume that the same features cannot be captured at every time step, which is more realistic, and this assumption increases the complexity of the scenarios considered in the paper.

For the considered scenarios, we are interested in investigating data-driven algorithms that can help determine the appropriate camera angles so that the maximum number of features are detected. In particular, this paper makes the following four contributions.

1. We design a reward function combining two components: the detection reward and the sinusoidal reward. The detection reward uses historical feature detection data to assign rewards to the chaser states. The second component utilizes the predictions of the feature locations to calculate the difference between the ideal and actual chaser states and assign rewards to the chaser states from the difference. Simulation results from the combined reward model show that the proposed algorithm can drive the chaser toward successful feature detection performance.
2. We create data-driven models based on the Gaussian Process (GP) regression for predicting the feature positions, which are utilized for the sinusoidal reward calculation. Due to the implementation of the GP, the proposed algorithm can be executed without requiring a physics model.
3. We implement the Fast Fourier Transform for appraising the target rotational period and use the estimated period as the initial guess of the periodicity hyper-parameter for training the GP models. This intelligent choice results in generating GP models with highly accurate prediction performance.
4. We utilize a GP for creating a reward distribution model over the chaser states. Using this model as the surrogate model and the Upper Confidence Bound (UCB) function as the acquisition function, we employ the Bayesian Optimization (BO) technique to determine the appropriate camera directional angles. We demonstrate that BO with the reward distribution model using a GP can determine the camera directional angles that lead to satisfactory feature detection performance.

The paper is structured as follows: Section 2 provides the detail of the proposed BO-based framework entitled Space Object Chaser-Resident Assessment Feature Tracking (SOCRAFT) for feature detection of the dynamic target in the 3D spatial domain, Section 3 presents the simulation results for the 2D and 3D scenarios to show the efficacy of the proposed algorithm, and Section 4 provides the concluding remarks.

## 2. Methodology

In this section, we present our proposed SOCRAFT algorithm that utilizes the Gaussian Process (GP) and Bayesian Optimization (BO) for determining the camera direction angles to detect the features of the target space object within the camera range and FOV. The problem specification along with the simulation scenario including the orbital model, the camera measurement model, and the reward models are provided in detail. Additionally, the procedures for predicting the feature locations as well as the target rotational period are explained. This section also highlights the overall BO-based SOCRAFT algorithm for feature detection of the target space object. Note that the description of the proposed SOCRAFT algorithm presented in this section is for the 3D spatial domain, but the algorithm can be implemented in the 2D domain with some minor changes.

### 2.1. Problem Specification in the 3D Spatial Domain

**Figure 1:**
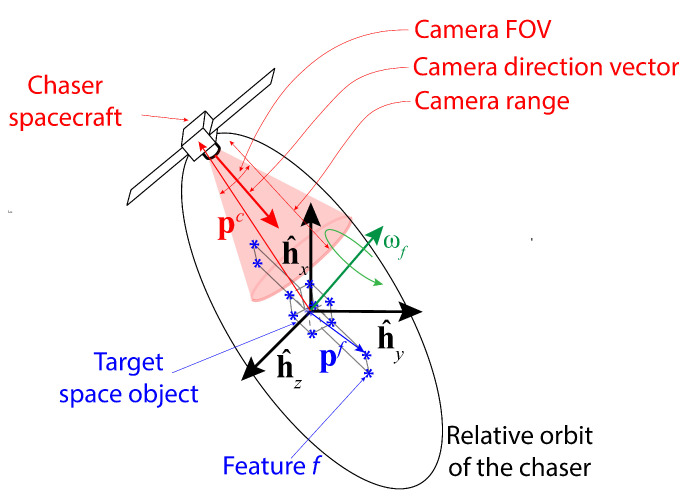
The schematic of the simulation setup; the black arrows represent the target Hill frame H, and the green arrow represents the target’s rotation axis. The closed chaser relative orbit around the target is shown as a black ellipse, and the blue asterisk (*) symbols indicate the constituent features of the target space object. A feature is assumed to be detected if it is located within the camera detection range and FOV, depicted by a red-shaded conic region.

The simulation scenario for this study is provided in Figure 1. A chaser carrying a monocular camera with limited range $d_{cam}$ and field of view (FOV) as well as a single-beam LIDAR orbits a target space object which consists of a known number of features, *M*. The chaser location $p^{c}\left(k\right)$ and the feature locations ${\left\{p^{f}\left(k\right)\right\}}_{f=1}^{M}$ at any time step *k* are defined in the target Hill frame H with the origin at the center of the mass of the target and the unit vectors $\left\{{\hat{h}}_{x},{\hat{h}}_{y},{\hat{h}}_{z}\right\}$. For simplicity, it is assumed that the orbits of the chaser and the target (or equivalently, the trajectories of the center of masses of both the chaser and the target) are known. Therefore, the target Hill frame is accurately known. Also, the target is assumed to rotate about a fixed axis with a rotational speed $\omega_{f}$ and period *P*, which are unknown constants for this study. The chaser orbit around the target is presumed to be a natural closed relative orbit which is energy-efficient and thus obtained from the solutions of the Clohessy–Wiltshire equations, given as follows [35]:

(1) $$\begin{array}{cc} x(t_{k}) & =A_{0}\cos\left(nt_{k}+\alpha \right) \end{array}$$

(2) $$\begin{array}{cc} y(t_{k}) & =-2A_{0}\sin\left(nt_{k}+\alpha \right)+y_{off} \end{array}$$

(3) $$\begin{array}{cc} z(t_{k}) & =B_{0}\cos\left(nt_{k}+\beta \right), \end{array}$$

where $x(t_{k})$, $y(t_{k})$, and $z(t_{k})$ are the components of $p^{c}\left(t_{k}\right)$ at time instance $t_{k}$, which corresponds to the *k*th time step, *n* denotes the orbital rate of the target, $A_{0}$, $B_{0}$, α, β, and $y_{off}$ are integral constants obtained from the initial conditions.

The chaser state $X^{c}\left(k\right)={\left[k,x\left(k\right),y\left(k\right),z\left(k\right),\theta \left(k\right),\psi \left(k\right)\right]}^{T}$ consists of time step *k*, the position components of the chaser location $p^{c}\left(k\right)={\left[x\left(k\right),y\left(k\right),z\left(k\right)\right]}^{T}$, and the camera directional angles {θ(k),ψ(k)} at that instance (ψ=0 in 2D and thus removed from consideration in 2D cases). The unit vector in the direction of the camera orientation or, in short, the camera direction vector $u_{cam}\left(k\right)={\left[u_{cam,x}\left(k\right),u_{cam,y}\left(k\right),u_{cam,z}\left(k\right)\right]}^{T}$, can be obtained from the following equation:

(4) $$u_{cam}\left(k\right)=\left[\begin{array}{c} \cos(\theta (k))\cos(\psi (k))\\ \sin(\theta (k))\cos(\psi (k))\\ \sin(\psi (k)) \end{array}\right]$$

Conversely, if the camera direction vector $u_{cam}\left(k\right)$ is known, then the camera directional angles {θ(k),ψ(k)} can be calculated using Equations (5) and (6).

(5) $$\begin{array}{cc} \theta (k) & =\arctan\left(\frac{u_{cam,y}\left(k\right)}{u_{cam,x}\left(k\right)}\right) \end{array}$$

(6) $$\begin{array}{cc} \psi (k) & =\arcsin(u_{cam,z}\left(k\right)) \end{array}$$

Note that in this study, ψ(k) is restricted between −90 and 90 degrees.

**Figure 2:**
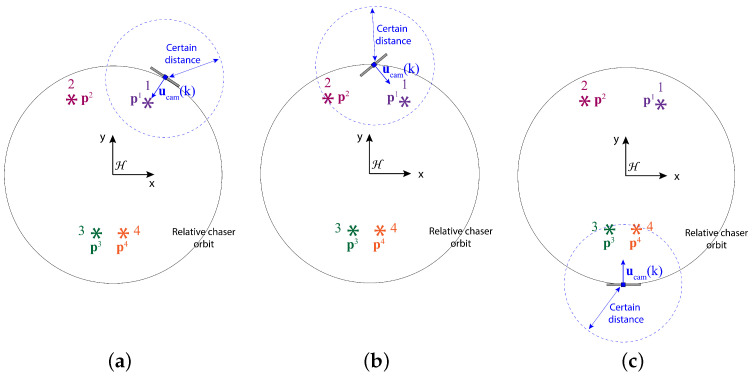
Illustration of the definition of the optimal camera direction angle θ for different scenarios in the 2D spatial domain. (**a**) Only one feature within a certain distance: the camera is pointed toward that feature; (**b**) two features within a certain distance and the features are not simultaneously detectable: the camera is pointed toward the closest feature; (**c**) two features within a certain distance and the features are simultaneously detectable: the camera is pointed in between the features.

The objective is to find the directional angles for the camera with a limited FOV and range so that most features within the camera range are successfully detected. In other words, we intend to find the camera directional angles {θ(k),ψ(k)} at any time step *k* as close as possible to the optimal camera directional angles $\left\{\theta_{opt}\left(k\right),\psi_{opt}\left(k\right)\right\}$ leading to the highest number of features detected. In this context, the optimal camera directional angles are defined as follows:

- The camera is turned straight toward a feature *f* or $\theta_{opt}\left(k\right)=\theta_{ideal}^{f}\left(k\right)$ and $\psi_{opt}\left(k\right)=\psi_{ideal}^{f}\left(k\right)$ if the rest of the features are further than a certain distance from the chaser (see Figure 2a). Here, $\theta_{ideal}^{f}\left(k\right)$ and $\psi_{ideal}^{f}\left(k\right)$ are the true ideal camera directional angles for feature *f* given that the camera direction vector exactly points toward feature *f*. The true ideal camera directional angles are defined as follows:
  (7) $$\begin{array}{cc} \theta_{ideal}^{f}\left(k\right) & =\arctan\left(\frac{p_{y}^{fc}\left(k\right)}{p_{x}^{fc}\left(k\right)}\right) \end{array}$$
  (8) $$\begin{array}{cc} \psi_{ideal}^{f}\left(k\right) & =\arcsin\left(\frac{p_{z}^{fc}\left(k\right)}{\left|\right|p^{fc}\left(k\right){\left|\right|}_{2}}\right), \end{array}$$
  where $p^{fc}\left(k\right)=\left[p_{x}^{fc}\left(k\right),p_{y}^{fc}\left(k\right),p_{z}^{fc}\left(k\right)\right]=p^{f}\left(k\right)-p^{c}\left(k\right)$ is the relative position of feature *f* with respect to the chaser, $0\leq \theta_{ideal}^{f}\left(k\right)\leq 360$ degrees, and $-90\leq \psi_{ideal}^{f}\left(k\right)\leq 90$ degrees.
- If two or more features are within the certain range, but they are not simultaneously detectable, the chaser should point the camera toward the closest feature (see Figure 2b).
- If two or more features are within the certain range and they are simultaneously detectable, the chaser should point the camera in such a direction so that the maximum number of features are detected (see Figure 2c). In this work, we assume the following definition of $\left\{\theta_{opt}\left(k\right),\psi_{opt}\left(k\right)\right\}$ for this case with two features *p* and *q*:
  (9) $$\begin{array}{cc} \theta_{opt}\left(k\right) & =\frac{1}{w_{d}^{p}+w_{d}^{q}}\left(w_{d}^{p}\theta_{ideal}^{p}\left(k\right)+w_{d}^{q}\theta_{ideal}^{q}\left(k\right)\right) \end{array}$$
  (10) $$\begin{array}{cc} \psi_{opt}\left(k\right) & =\frac{1}{w_{d}^{p}+w_{d}^{q}}\left(w_{d}^{p}\psi_{ideal}^{p}\left(k\right)+w_{d}^{q}\psi_{ideal}^{q}\left(k\right)\right), \end{array}$$
  where $w_{d}^{p}$ and $w_{d}^{q}$ are distance weights associated with features *p* and *q*, and the weights are calculated based on the distances between the features and the chaser, $d^{pc}$ and $d^{qc}$. Due to these weights, the optimal camera directional angles are more inclined toward the ideal camera directional angles of the feature within the certain range that is closer to the chaser. How to design the distance-based weight is provided in Section 2.3.2.

### 2.2. Camera Measurement Model

Feature *f* is considered to be detected if the following conditions are satisfied.

(11) $$d^{fc}\left(k\right)\leq d_{cam}\;\&\;\Delta \Theta^{f}\left(k\right)\leq \frac{1}{2}FOV,$$

where $d^{fc}\left(k\right)=\left|\right|p^{fc}\left(k\right){\left|\right|}_{2}$ is the distance between the chaser and feature *f*, and $\Delta \Theta^{f}\left(k\right)$ is the true angle between the camera direction vector $u_{cam}\left(k\right)$ and the position vector of the feature relative to the chaser, $p^{fc}\left(k\right)$, at time step *k*. Together, these detection criteria state that in order to be detected, a feature needs to be located within the camera FOV and the camera range.

As mentioned before, in this study, we assume that the chaser is equipped with a monocular camera and a single-beam LIDAR sensor. From the images captured by the camera and the noisy range measurements obtained from the LIDAR, it is possible to determine the noisy 3D locations of the detected features [32]. Additionally, there exist a number of methods for feature detection, such as SIFT [36], SURF, AKAZE, ORB [37], etc. In this work, we do not implement feature detection methods and assume that the noisy 3D locations of the particular features are available from image processing algorithms.

If feature *f* is detected, then the observed noisy feature location ${\tilde{p}}^{f}\left(k\right)$ from the camera measurement model is obtained from the following equation:

(12) $${\tilde{p}}^{f}\left(k\right)=p^{f}\left(k\right)+w\left(k\right),$$

where $w\left(k\right)\in R^{3}$ is the white noise with the components $\left(w_{x},w_{y},w_{z}\right)∼N\left(0,\sigma_{m}\right)$, where $\sigma_{m}$ is the standard deviation of the noise components. Note that the 3D positions extracted from the captured images and range measurements are usually expressed in the camera frame. However, based on the assumptions of the camera directional angles and the centers of mass of the target and chaser being known, the noisy 3D positions of the features can be easily transformed into the H frame. Hence, we skip this trivial transformation process and directly assume that the 3D noisy feature positions are expressed in the H frame. We also assume that the camera measurement model returns the index *f* of the detected feature. Therefore, the output from the camera measurement model Y(k) contains the following information: time step *k*, the corresponding noisy observed location of the detected feature ${\tilde{p}}^{f}\left(k\right)$, and the detected feature index *f*. The outputs from the camera measurement model are stored in a data set $D_{cam}$, which has the format as provided in Table 1 where f(1), f(2), ⋯, and f(k) denote the indices of the detected features at time steps 1,2,⋯,k. If multiple features are detected simultaneously, the measurement associated with each detected feature is added separately in $D_{cam}$. Furthermore, if none of the features are detected, no measurement information is stored in $D_{cam}$.

### 2.3. Reward Model

Each chaser state $X^{c}\left(k\right)$ is assigned a scalar reward r(k) that is the sum of two components: the feature detection reward $r_{det}\left(k\right)$ and the sinusoidal reward $r_{sin}\left(k\right)$. The details of the feature detection reward, the sinusoidal reward, and the combined reward r(k) are provided below.

#### 2.3.1. Feature Detection Reward ($r_{det}$)

The feature detection reward $r_{det}\left(k\right)$ is assigned to the chaser state $X^{c}\left(k\right)$ based on whether features have been detected by the camera from this chaser state or not. If the total number of detected features by the camera is $M_{det}\left(k\right)$, then a constant reward $r_{max}$ is assigned to the chaser state for each detected feature, and the assigned feature detection reward $r_{det}\left(k\right)$ is obtained from the following equation:

(13) $$r_{det}\left(k\right)=w_{det}r_{max}M_{det}\left(k\right),$$

where $w_{det}$ is a constant weight for adjusting the priority of the detection reward.

Since this reward model solely depends on the feature detection and is discrete in nature, it does not lead to the optimal camera directional angles. For instance, consider that two sets of camera directional angles $\left\{\theta_{1}\left(k\right),\psi_{1}\left(k\right)\right\}$ and $\left\{\theta_{2}\left(k\right),\psi_{2}\left(k\right)\right\}$ can lead to the detection of feature *f* from the same chaser location $p^{c}\left(k\right)$ at time step *k*. Thus, both of the corresponding chaser states $X_{1}\left(k\right)=\left[k,p^{c}\left(k\right),\theta_{1}\left(k\right),\psi_{1}\left(k\right)\right]$ and $X_{2}\left(k\right)=\left[k,p^{c}\left(k\right),\theta_{2}\left(k\right),\psi_{2}\left(k\right)\right]$ receive the same $r_{det}$. Therefore, the true $r_{det}$ distribution over the feasible domain of {θ,ψ} at a certain location and time is flat, and consequently, it is not possible to determine the optimal camera direction angles with only this reward even with a considerably large data set. Additionally, the chaser is biased toward selecting the camera directional angles based on the feature detection at historical chaser states. Since it cannot be guaranteed that the features are always detected, the reward model cannot successfully detect the features even when they are within the detection range unless a very big data set is provided. Due to these issues associated with the feature detection reward, we designed the sinusoidal reward model.

#### 2.3.2. Sinusoidal Reward ($r_{sin}$)

Contrary to the feature detection reward, which depends only on the historical detection performance, the sinusoidal reward is to be calculated from the difference between the ideal and actual chaser states. Specifically, as the chaser locations are predefined, this reward is determined primarily by the angle between the ideal camera direction for a specific feature and the actual camera direction vector. This reward is named the ‘sinusoidal reward’ because of the presence of the cosine half of this angle in the expression of this reward. In this context, the ideal camera direction vector at time step *k* for any feature *f* is the unit vector in the direction of the relative position vector of the feature with respect to the chaser, $p^{fc}\left(k\right)$. Assuming that only the estimated location of the feature, ${\hat{p}}^{f}\left(k\right)$, is available from a prediction model, the estimated relative position of feature *f* with respect to the chaser, ${\hat{p}}^{fc}\left(k\right)$, can be calculated from the following equation:

(14) $${\hat{p}}^{fc}\left(k\right)={\hat{p}}^{f}\left(k\right)-p^{c}\left(k\right)$$

**Figure 3:**
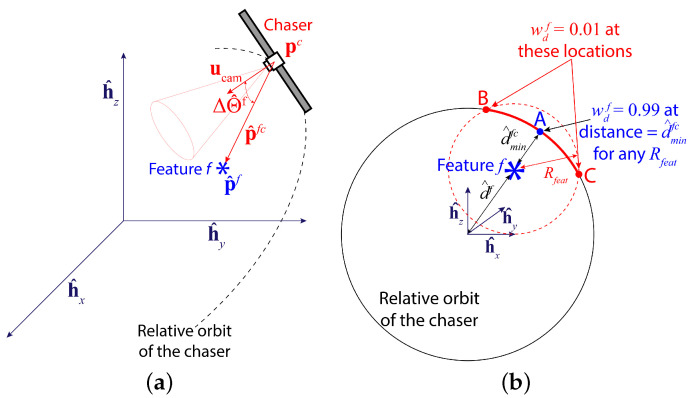
Illustrations showing (**a**) the estimated camera directional angle difference $\Delta {\hat{\Theta }}^{f}$, which is the angle between $u_{cam}$ and ${\hat{p}}^{fc}$, and (**b**) the region of influence of a feature *f*, which is a spherical region around the feature centered at the feature location and a radius $R_{feat}$.

Then, the estimated camera directional angle difference $\Delta {\hat{\Theta }}^{f}\left(k\right)$ is the angle between $u_{cam}\left(k\right)$ and ${\hat{p}}^{fc}\left(k\right)$ as shown in Figure 3a, and the angle is expressed as:

(15) $$\Delta {\hat{\Theta }}^{f}\left(k\right)=\arccos\left(\frac{{\hat{p}}^{fc}\left(k\right)\cdot u_{cam}\left(k\right)}{\left|\right|{\hat{p}}^{fc}\left(k\right)\left|\right|}\right)$$

**Figure 4:**
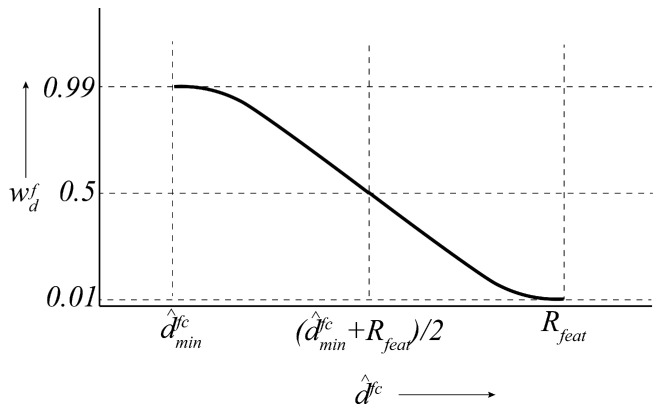
The reverse sigmoid function for obtaining the distance weight $w_{d}^{f}$ for any feature *f*.

The sinusoidal reward assigned to the chaser state $X^{c}\left(k\right)$ due to feature *f*, $r_{sin}^{f}\left(k\right)$ and the total sinusoidal reward $r_{sin}\left(k\right)$ for all features are then obtained from the following equations:

(16) $$r_{sin}^{f}\left(k\right)=w_{d}^{f}r_{max}{\left[\cos\left(\frac{\Delta {\hat{\Theta }}^{f}\left(k\right)}{2}\right)\right]}^{2}$$

(17) $$r_{sin}\left(k\right)=\sum_{f=1}^{M}r_{sin}^{f}\left(k\right),$$

where $w_{d}^{f}$ is the distance weight for regulating the influence of feature *f* on the total sinusoidal reward $r_{sin}\left(k\right)$ depending on $d^{fc}$. In other words, including the weight $w_{d}^{f}$ in the sinusoidal reward model encourages the chaser to detect the closest features and ignore the furthest ones. To that purpose, we define a parameter $R_{feat}$ such that any feature *f* contributes to or influences $r_{sin}$ ($r_{sin}^{f}>0$) if $d^{fc}<R_{feat}$, otherwise, the feature has almost no influence on $r_{sin}$ ($r_{sin}^{f}\approx 0$). This is shown in Figure 3b, where feature *f* has a spherical region of influence, centered at its current position with a radius $R_{feat}$. The chaser is the closest to this feature at point A. The furthest points within the feature’s region of influence are points B and C, where the chaser relative orbit and the boundary of the influence region intersect. The distance weight $w_{d}^{f}$ is defined in a way so that when the chaser is located at point B or C, $w_{d}^{f}=0.01$ and at point A, $w_{d}^{f}=0.99$. Furthermore, $0.01\leq w_{d}^{f}\leq 0.99$ if the chaser is within the feature’s region of influence (the solid red arc). This weight is defined as a reverse sigmoid function as shown in Figure 4 and mathematically written as follows:

(18) $$w_{d}^{f}={\left[1+\exp\left(\frac{2\ln(99)}{R_{feat}-{\hat{d}}_{min}^{fc}}\left({\hat{d}}^{fc}\left(k\right)-\frac{R_{feat}+{\hat{d}}_{min}^{fc}}{2}\right)\right)\right]}^{-1},$$

where ${\hat{d}}_{min}^{fc}$ and ${\hat{d}}^{fc}\left(k\right)=\left|\right|{\hat{p}}^{fc}\left(k\right)\left|\right|$ are the estimated minimum and current distances between the chaser and feature *f* at time step *k*. The derivation of Equation (18) is provided in Appendix A.

To understand the reason behind the design of this reward model, consider the following example. Assume that the chaser is orbiting a target with only two features, *f* and *g*. With the progression of time, the chaser approaches feature *f* first and then feature *g* as it gets away from feature *f* and so on. When the chaser is close to feature *f* such that $d^{fc}<R_{feat}$ and $d^{gc}>R_{feat}$, only this feature influences $r_{sin}$ because $w_{d}^{f}>0.01$ and $w_{d}^{g}\approx 0$. In this scenario, the chaser can maximize $r_{sin}$ by pointing the camera directly toward feature *f* ($\theta \approx \theta_{ideal}^{f}$), resulting in a small camera directional angle difference $\Delta \Theta^{f}$. As the chaser travels toward feature *g* from feature *f*, $w_{d}^{f}$ decreases as $w_{d}^{g}$ rises. If $d^{gc}<R_{feat}$, feature *g* starts to influence $r_{sin}$ and hence, for achieving the maximum $r_{sin}$, the chaser starts to turn the camera toward feature *g* gradually. During this transition of feature influence on $r_{sin}$, the camera is pointed toward somewhere in between these two features. At this stage, the features may or may not be detected given the positions of the feature positions, the chaser trajectory, the camera range, and the FOV. If $d^{fc}>R_{feat}$, then feature *f* does not influence $r_{sin}$ anymore, and the camera should be pointed straight toward feature *g* in the process of minimizing $\Delta \Theta^{g}$. In summary, with the sinusoidal reward model, we expect to see very small $\Delta \Theta^{f}$ if feature *f* is the only feature within $R_{feat}$ from the chaser, and during the movement of the chaser from one feature to another, the camera is slowly turned from the first feature toward the second one.

#### 2.3.3. Combined Reward (*r*)

The combined reward *r* is the sum of the feature detection reward $r_{det}$ and the sinusoidal reward $r_{sin}$ and written as follows:

(19) $$r\left(k\right)=r_{det}\left(k\right)+r_{sin}\left(k\right)=w_{det}r_{max}M_{det}\left(k\right)+\sum_{f=1}^{M}w_{d}^{f}r_{max}{\left[\cos\left(\frac{\Delta {\hat{\Theta }}^{f}\left(k\right)}{2}\right)\right]}^{2}$$

The combined reward model takes advantage of both the sinusoidal and the feature detection rewards by overcoming one reward’s drawback by the other. For example, suppose a feature was detected in the past from a chaser location with a particular camera angle. In that case, the chaser is biased toward selecting a similar camera angle from a nearby location. This is because the previous chaser state at which the feature was detected was assigned $r_{det}=w_{det}r_{max}>0$, and as a result, $r_{det}$ will affect the combined reward *r* as well as the camera directional angles. On the contrary, if no feature was detected in the past from a chaser location, the corresponding chaser states have zero $r_{det}$, and hence, $r_{sin}$ is dominant in this scenario. Consequently, for a nearby feature, the camera directional angles will reach close to the ideal camera direction angles ($\Delta \Theta^{f}\approx 0$). In this way, the combined reward model overcomes the drawback of the feature detection reward and leads to the detection of the features irrespective of whether the features are detected from the current chaser position or were detected in the past. However, we should observe fluctuations in the camera directional angles {θ,ψ} and the camera directions angle differences ${\left\{\Delta \Theta^{f}\right\}}_{f=1}^{M}$ due to the varying influences of $r_{det}$ and $r_{sin}$ on *r*. Additionally, the disadvantage of the sinusoidal reward comes from scenarios where multiple features are within $R_{feat}$ from the chaser but not simultaneously detectable. In these scenarios, the chaser will point the camera between the features and may be unable to detect any of them for some time with only $r_{sin}$. Due to the bias toward certain camera directional angles resulting from $r_{det}$, the chaser has greater chances of detecting either of the nearby features in these scenarios.

The formal algorithm of the Reward() function to assign rewards to the chaser states is provided in Algorithm 1.
**Algorithm 1:** Working mechanism of the Reward() function
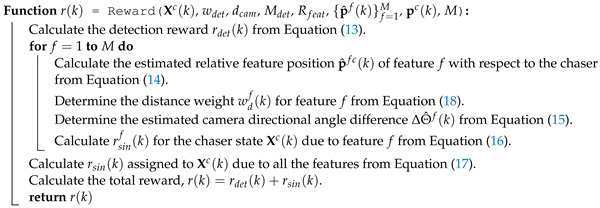


### 2.4. Gaussian Process (GP) Models

To accomplish the task of obtaining the camera directional angles, we generate GP models to fulfill two objectives: estimating the feature positions (GP-feature models) and modeling the reward distribution over the chaser states (GP-reward model).

#### 2.4.1. GP-Feature Models

The sinusoidal reward is designed based on the assumption that the estimated locations of all features ${\left\{{\hat{p}}^{f}\left(k\right)=\left[{\hat{p}}_{x}^{f}\left(k\right),{\hat{p}}_{y}^{f}\left(k\right),{\hat{p}}_{z}^{f}\left(k\right)\right]\right\}}_{f=1}^{4}$ at any time step *k* are available. Therefore, we need to create prediction models for the feature locations throughout the entire duration of the mission from the camera observations, and we utilize the GP-feature models for this task.

For any feature *f*, three separate single-output GP-feature models are created to model the feature position components ${\left\{p_{i}^{f}\right\}}_{i=1}^{3}=\left\{p_{x}^{f},p_{y}^{f},p_{z}^{f}\right\}$ from past observations of the feature stored in the data set $D_{cam}$. Thus, if the target’s total number of features is *M*, a total of 3M GP-feature models are created (2M in 2D). The GP-feature prediction model of the *i*th spatial dimension component $p_{i}^{f}$ is denoted by $\Gamma_{i}^{f}\left(k,{\tilde{p}}_{i}^{f}\right)$, where i=1,2,3 and f=1,2,⋯,M. The two-dimensional training data set $D_{i}^{f}$ for $\Gamma_{i}^{f}\left(k,{\tilde{p}}_{i}^{f}\right)$ consists of the past time steps which correspond to the detection of feature *f* and the associated *i*th position components ${\tilde{p}}_{i}^{f}$ of ${\tilde{p}}^{f}$. If the features are stationary, the Matérn3/2 or the constant kernel for GP can create $\Gamma_{i}^{f}\left(k,{\tilde{p}}_{i}^{f}\right)$ with acceptable accuracy. However, if the features are dynamic and rotating, then the standard periodic kernel [38,39] is suitable for this task due to the periodic nature of the feature motion. The mathematical expression of the periodic kernel is provided below:

(20) $$K_{f}\left(k_{i},k_{j}\right)=\sigma_{k}^{2}\cdot \exp\left(-\frac{2}{l_{k}^{2}}\sin^{2}\left(\frac{\pi \delta k}{P}\right)\right),$$

where $\delta k=|k_{i}-k_{j}|$. Also, $l_{k}$, $\sigma_{k}$, and *P* are the length-scale, smoothness, and periodicity hyper-parameters.

The standard periodic kernel requires the periodicity hyper-parameter to create a reliable GP model of an underlying periodic function. The periodicity is determined from the maximum likelihood estimation method, which is highly influenced by the initial estimation of the period the user provides. An initial value close to the appropriate period helps reduce the computation time to properly obtain the periodicity hyper-parameter that fits the provided data. Therefore, we use the Fast Fourier Transform (FFT) algorithm to estimate the rotational period of the target. First, the initial GP-feature model $\Gamma_{i}^{f}\left(k,{\tilde{p}}_{i}^{f}\right)$ for the *i*th component of feature *f* position is created using the Matérn3/2 kernel for estimating the feature locations at any given instance. Next, the mean predicted ${\hat{p}}_{i}^{f}$ are sampled from $\Gamma_{i}^{f}\left(k,{\tilde{p}}_{i}^{f}\right)$ at constant time intervals. Then, the FFT is implemented on the samples, and the frequency $F_{max}$ with the highest magnitude is obtained. Afterward, the estimated period ${\hat{P}}_{i}^{f}$ determined from the *i*th position component of feature *f* is obtained as the inverse of $F_{max}$. These sequences of operations are conducted for each feature and each spatial dimension component, a total of 3M number of times, and the final estimated target rotational period $\hat{P}$ is determined as the average of ${\left\{{\left\{P_{i}^{f}\right\}}_{i=1}^{3}\right\}}_{f=1}^{M}$. Once $\hat{P}$ is available, the GP-feature models ${\left\{{\left\{\Gamma_{i}^{f}\left(k,{\tilde{p}}_{i}^{f}\right)\right\}}_{i=1}^{3}\right\}}_{f=1}^{M}$ can be recreated using the periodic kernel. The formal algorithm of the function findFeaturePeriod() that determines the estimated rotational period is provided in Algorithm 2.
**Algorithm 2:** Working mechanism of the findFeaturePeriod() function
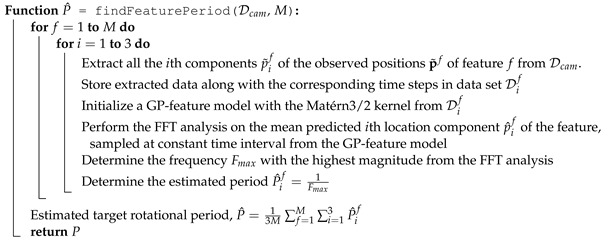


#### 2.4.2. GP-Reward Model

The GP-reward model Λ(k,x,y,z,θ,ψ,r) or simply Λ is defined as the prediction model for estimating the distribution of reward *r* over the chaser state $X^{c}\left(k\right)=\left[k,x,y,z,\theta ,\psi \right]$. The training data set $D_{r}$ for this model can have the maximum size *S* and consists of seven dimensions: time step *k*, the chaser position components x,y,z, the camera directional angles θ,ψ, and the reward *r* assigned to the chaser state. The format of the data set $D_{r}$ is shown in Table 2.

We utilize a kernel for the GP-reward model that is a product of four fundamental kernels: a 1D periodic kernel for the temporal dimension with period $\hat{P}$, a 3D Matérn3/2 kernel for the three spatial dimensions, a 1D periodic kernel for θ with period 2π radians, and a 1D Matérn3/2 kernel for ψ. Mathematically, the kernel is written as follows:

(21) $$\begin{array}{cc} K(X_{i}^{c},X_{j}^{c}) & =K_{1}\left(k_{i},k_{j}\right)\cdot K_{2}\left(p_{i}^{c},p_{j}^{c}\right)\cdot K_{3}\left(\theta_{i},\theta_{j}\right)\cdot K_{4}\left(\psi_{i},\psi_{j}\right)\\ K_{1}\left(k_{i},k_{j}\right) & =\sigma_{k}^{2}\cdot \exp\left(-\frac{2}{l_{k}^{2}}\sin^{2}\left(\frac{\pi \delta k}{P}\right)\right)\\ K_{2}\left(p_{i}^{c},p_{j}^{c}\right) & =\sigma_{p^{c}}^{2}\cdot \left(1+\frac{\sqrt{3}\delta p^{c}}{l_{p^{c}}}\right)\exp\left(-\frac{\sqrt{3}\delta p^{c}}{l_{p^{c}}}\right)\\ K_{3}\left(\theta_{i},\theta_{j}\right) & =\sigma_{\theta }^{2}\cdot \exp\left(-\frac{2}{l_{\theta }^{2}}\sin^{2}\left(\frac{\pi \delta \theta }{2\pi }\right)\right)\\ K_{4}\left(\psi_{i},\psi_{j}\right) & =\sigma_{\psi }^{2}\cdot \left(1+\frac{\sqrt{3}\delta \psi }{l_{\psi }}\right)\exp\left(-\frac{\sqrt{3}\delta \psi }{l_{\psi }}\right), \end{array}$$

where $X^{c}={\left[k,x,y,z,\theta ,\psi \right]}^{T}$, $p^{c}={\left[x,y,z\right]}^{T}$, $\delta k=|k_{i}-k_{j}|$, $\delta p^{c}=\left|\right|p_{i}^{c}-p_{j}^{c}{\left|\right|}_{2}$, $\delta \theta =|\theta_{i}-\theta_{j}|$, and $\delta \psi =|\psi_{i}-\psi_{j}|$. Also, $l_{k}$, $l_{p^{c}}$, $l_{\theta }$, and $l_{\psi }$ are the length-scale hyper-parameters, $\sigma_{k}$, $\sigma_{p^{c}}$, $\sigma_{\theta }$, and $\sigma_{\psi }$ are the hyper-parameters to regulate the smoothness of the underlying functions, and *P* is the periodicity hyper-parameter of $K_{1}$. These hyper-parameters are approximated by performing the optimization of the maximum likelihood [38].

### 2.5. Limitations of the GP Models

The GP is regarded as a highly capable non-parametric regression technique in statistical and ML applications, but it suffers from several disadvantages. The main disadvantage of the GP comes from the associated computational complexity. The computational complexity of a GP model with a data set of size *n* is $O(n^{3})$, and the storage requirement is $O(n^{2})$ [38]. Due to the high computation complexity of GP models with large training data sets, the GP-reward model Λ in our study is expensive to compute since the data set size is on the order of thousands, unlike the GP feature models $\Gamma_{i}^{f}$ with a few hundred data points. However, there exist approximation methods to reduce the computational complexity. For instance, the Sparse Gaussian Process (SGP) reduces the computational complexity to $O(m^{2}n)$ with m<n being the number of inducing variables, but this reduction comes at the expense of the prediction accuracy [38].

Another drawback associated with the GP is the selection of the kernel function. To effectively learn important patterns between the input and output training variables, the choice of kernel is of major importance. However, there is no fixed guideline on how to select a kernel. Finding the appropriate kernel is an arduous task and may require one to go through numerous trials and errors to manually find the right kernel for the corresponding data set.

Additionally, the prediction performance of a GP model greatly relies on the hyper-parameters, which usually are obtained from the maximum likelihood optimization. Local optimal solutions might be selected as the hyper-parameters, which will result in poor prediction performance. To avoid this issue, intelligent choices of the initial guesses of the hyper-parameters or multiple executions of the optimization operation with randomly selected initial guesses are needed. For the purpose of obtaining the initial guess of the periodicity hyper-parameter intelligently, we implemented the FFT analysis in this paper.

### 2.6. Bayesian Optimization (BO)

**Figure 5:**
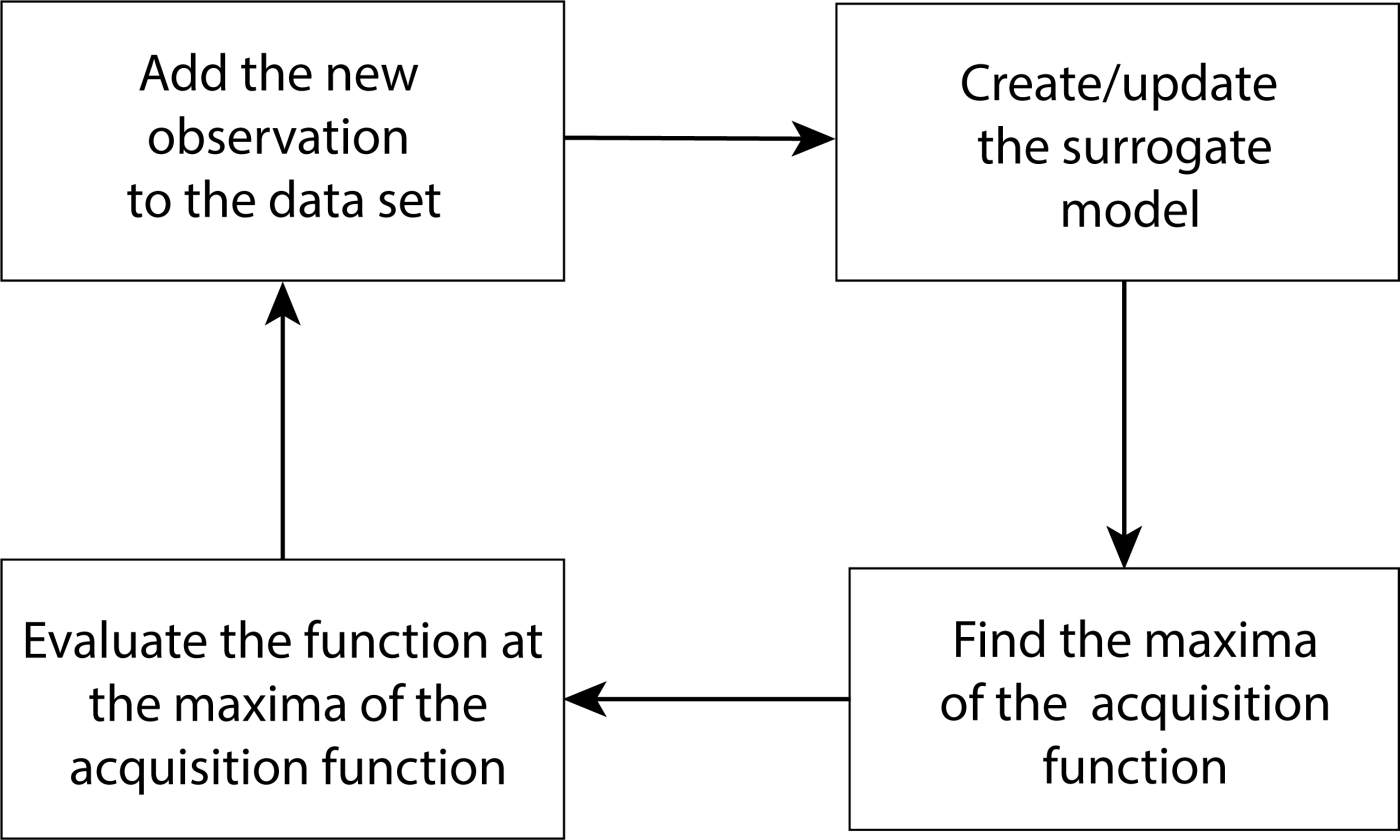
Bayesian Optimization framework.

BO is an optimization algorithm to solve for the global maxima of a black-box function or a function with a high computational cost. The main components of BO are a probabilistic surrogate model and an acquisition function. As shown in Figure 5, BO executes the following steps to sequentially reach the global maxima of the underlying function. First, a surrogate model is created from existing data set/information regarding the underlying function. Second, the acquisition function is evaluated from the surrogate model and the maxima of the acquisition function is obtained from optimization. Third, the function is evaluated at the maxima of the acquisition function. Finally, the new observation is added to the data set, followed by updating the surrogate model.

Popular acquisition functions include the Upper Confidence Bound (UCB) function, the Probability of Improvement (PI) function, and the Expected Improvement (EI) function. For this work, we chose the UCB function as the acquisition function because it provides a simple way to regulate exploration and exploitation. The UCB function has the following expression:

(22) $$\alpha \left(X^{c},\kappa \right)=\mu \left(X^{c}\right)+\kappa \left(\sigma \left(X^{c}\right)\right),$$

where $\mu (X^{c})$ and $\sigma (X^{c})$ are the mean and the standard deviation over the input domain $X^{c}$ obtained from the surrogate model, and κ is the parameter for regulating the prioritization between exploration and exploitation.

Setting a large value for κ indicates that the locations in $X^{c}$ with high σ values or uncertainty will be selected for sampling and BO looks for the maxima in an exploratory manner. On the contrary, a small value for κ indicates that the underlying function will be evaluated at locations where $\mu (X^{c})$ has larger values. As a result, BO is discouraged from exploring $X^{c}$ and realizes the exploitation of the current knowledge about the underlying function to find the global maxima.

In this work, the BO algorithm utilizes the GP-reward model Λ as the surrogate model and optimizes the UCB acquisition function $\alpha (X^{c},\kappa )$ to determine the camera directional angles. The optimization operation is written as follows:

(23) $$a^{*}=\underset{a\in A}{arg max}\alpha \left(X^{c},\kappa \right)=\underset{a\in A}{arg max}\left[\mu \left(X^{c}\right)+\kappa \sigma \left(X^{c}\right)\right],$$

where $a^{*}={\left[\theta^{*},\psi^{*}\right]}^{T}$ is the optimal action according to the BO in the action space A with the camera directional angles $\theta^{*}$ and $\psi^{*}$. Also, $\mu (X^{c})$ and $\sigma (X^{c})$ are the mean and standard deviation of the reward *r* obtained from Λ at any arbitrary chaser state. Equation (23) is solved by using a global optimizer, the ‘Basinhopping’ algorithm, which also requires a local optimizer for which we utilized the SLSQP algorithm. Note that the actions determined by BO were assumed to be instantaneously and exactly executed since camera attitude maneuver strategy was not considered in this study.

### 2.7. Simulation Workflow

The complete algorithm for the Space Object Chaser-Resident Assessment Feature Tracking (SOCRAFT) methodology is provided in Algorithm 3. The overall mission scenario can be divided into two stages: (i) the initial data collection and model generation stage and (ii) the BO implementation stage. The mission starts by initializing the relative chaser orbit ${\left\{p_{k}^{c}\right\}}_{k=1}^{T}$ from the initial time step k=1 till the end of mission k=T, the number of time steps for initial data collection $T_{init}$, the camera FOV and range $d_{cam}$, the maximum reward $r_{max}$, the feature detection reward weight $w_{det}$, and the radius of influence $R_{feat}$. The data set $D_{cam}$ for storing the camera measurements, $D_{chs}$ for storing the chaser states, and the training data set $D_{r}$ for the GP-reward model with the maximum size *S* are also initialized.

During the initial data collection and model generation stage, from time step k=1 to $k=T_{init}$, the chaser follows the predefined relative orbit trajectory while the camera directional angles {θ(k),ψ(k)} are randomly selected from uniform distributions with ranges 0≤θ≤360 degrees, −90≤ψ≤90 degrees. The chaser states $X^{c}\left(k\right)$ and the corresponding camera measurements Y(k) are stored in $D_{chs}$ and $D_{cam}$, respectively. Once the initial data are collected, the estimated target rotational period $\hat{P}$ is determined from the findFeaturePeriod() function if the features are rotating about a fixed axis ($\omega_{f}\neq 0$); otherwise, this step is skipped. Next, the GP-feature models ${\left\{{\left\{\Gamma_{i}^{f}\left(k,{\tilde{p}}_{i}^{f}\right)\right\}}_{i=1}^{3}\right\}}_{f=1}^{M}$ are initialized with either the Matérn3/2 ($\omega_{f}=0$) or the periodic kernel ($\omega_{f}\neq 0$). Afterward, the rewards ${\left\{r\left(k\right)\right\}}_{k=1}^{T_{init}}$ for all previous chaser states ${\left\{X^{c}\left(k\right)\right\}}_{k=1}^{T_{init}}$ are calculated from the Reward() function and the chaser states with their corresponding rewards are stored in $D_{r}$. The final step of the first stage is initializing the GP-reward model Λ from the data set $D_{r}$.
**Algorithm 3:** Bayesian Optimization-based SOCRAFT algorithm for detecting features of a non-cooperative and rotating targetInitialize chaser trajectory ${\left\{p_{k}^{c}\right\}}_{k=1}^{T}$, total step *T*, total steps for initial data collection $T_{init}$, camera FOV, camera range $d_{cam}$, maximum reward $r_{max}$, detection reward weight $w_{det}$, radius of influence $R_{feat}$, total number of features *M* Initialize data sets $D_{r}$ (with the maximum size *S*), $D_{cam}$, and $D_{chs}$ 
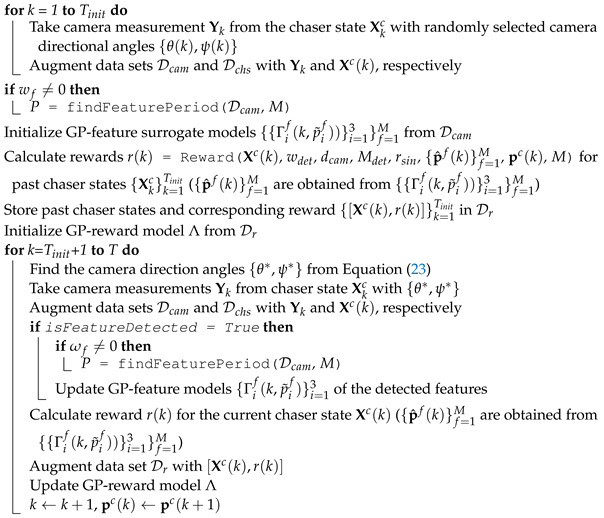


In the second stage of the mission, from $k=T_{init}+1$ to k=T, BO is implemented, and the camera directional angles are obtained from Equation (23). Then, the chaser points the camera in the direction of $u_{cam}\left(k\right)$ defined by the camera directional angles and takes camera measurement Y(k), which is stored in the data set $D_{cam}$. If the camera detects any feature (isFeatureDetected=True), the GP-feature models of the detected features are updated, and $\hat{P}$ is also updated from the findFeaturePeriod() function for dynamic features. Afterwards, the reward r(k) is calculated for the current chaser state $X^{c}\left(k\right)$ and $\left[X^{c}\left(k\right),r\left(k\right)\right]$ is stored in the data set $D_{r}$. Finally, the GP-reward model Λ is updated, and the chaser proceeds to the next predefined position. If k>S, then the oldest data point is removed from $D_{r}$ to accommodate the latest data point.

## 3. Results and Discussion

To demonstrate the effectiveness of the Space Object Chaser-Resident Assessment Feature Tracking (SOCRAFT) algorithm for detecting the features of a non-cooperative and dynamic target, we conducted simulations in both 2D and 3D spatial domains with the sinusoidal and the combined (feature detection + sinusoidal) rewards, and the results from the simulations are presented in this section. We provide the results obtained from the simulations in the 2D spatial domain alongside the 3D case because the 2D case is a more straightforward scenario and thus provides an easier way to understand the underlying working mechanisms of the proposed algorithm and its performance. As a reminder, the 2D case is a special scenario of the proposed algorithm and hence, the algorithm can be implemented in the 2D case with some minor adjustments.

All results presented in this section were obtained by conducting simulations on a desktop computer with 11th Gen Intel^®^ Core^TM^ i7-11700 (2.50 GHz), manufactured by Intel Corporation, Santa Clara, CA, USA and 16 GB of RAM.

### 3.1. Common Simulation Setup

**Figure 6:**
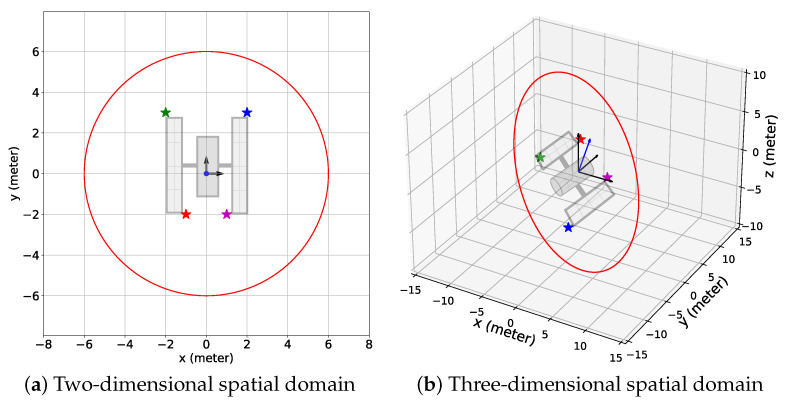
The simulation setups for the (**a**) 2D and (**b**) 3D spatial domains. Each setup consists of 4 features (star symbols) representing different corners of the solar panels of the target space objects (defunct satellites) and a circular relative chaser orbit (red closed curve). Features 1, 2, 3, and 4 are represented by the blue, green, red, and magenta stars, respectively. The black arrows indicate the axes of the target Hill frame H and in (**b**), the blue arrow denotes the target’s rotation axis.

Similar simulation scenarios were considered for the 2D and 3D cases, which consisted of a target with four features and a circular relative chaser orbit with the center positioned at the origin of the target Hill frame H, as shown in Figure 6. Note that although the elliptical relative orbits are a more practical choice, such orbits will vary the feature-chaser relative distance by a large margin. Consequently, for a short camera range, the chaser might be so far from the features that no feature is within the camera range at some point in time. On the contrary, a circular orbit gives the chaser the opportunity to be in the vicinity of the features most of the time, and a circular relative orbit also provides a simple simulation scenario, which is why we considered the relative chaser orbits to be circular in this study. However, any appropriate predefined relative chaser orbit is compatible with the proposed algorithm. The features were placed such that the chaser proceeded toward feature 1 first, then moved toward feature 2, followed by feature 3, and then feature 4, as the chaser followed the predefined trajectory. The duration for the chaser to complete one orbit along the predefined relative orbit was 180 time steps. Also, the standard deviation $\sigma_{m}$ for the components of the added noise w(k) in the camera measurement model was 0.3 m. Additionally, the parameter κ in Equation (23) was zero because we focused on investigating how well the proposed algorithm exploited the provided data given a considerably large data set. Moreover, $R_{feat}=1.25d_{cam}$ and for the simulations with the combined reward, $w_{det}=0.5$. The target rotational period *P* was assumed to be 900 time steps. Finally, the BO implementation stage duration, $T-T_{init}$, was 225 time steps or 1.25 chaser orbits. We chose this duration for the BO implementation stage so that the chaser had sufficient time to detect all features, and the detection time window was similar for all features considering the rotation of the target.

In this study, we used GPy [40] and GPflow [41], two Python-based open-source Gaussian Process frameworks for generating the GP-reward and GP-feature models, respectively. We utilized GPy to develop the GP-reward model because this model takes longer to be trained, and GPy offers faster computational capability than GPflow. On the contrary, GPflow was used for the GP-feature models since this package provides more reliable models. The GP-feature models were much quicker to be trained than the GP-reward model because of the smaller number of dimensions and smaller size of the training data sets.

### 3.2. Two-Dimensional Case

#### 3.2.1. Simulation Setup

For the 2D case, the initial feature positions (k=1) in the H frame were given as follows: $p^{1}\left(1\right)={\left[2,3\right]}^{T}$ meters, $p^{2}\left(1\right)={\left[-2,3\right]}^{T}$ meters, $p^{3}\left(1\right)={\left[-1,-2\right]}^{T}$ meters, and $p^{4}\left(1\right)={\left[1,-2\right]}^{T}$ meters. The features rotated with respect to the unit vector in the *z* direction, ${\hat{h}}_{z}$. The radius of the relative chaser orbit was 6 meters, and the chaser traveled along the trajectory in the counter-clockwise direction viewed from the top. Note that the 2D orbits on the xy plane derived from Equations (1)–(3) were always elliptical, but we considered the circular orbits on the xy plane for the sake of simplicity. Additionally, the camera range was $d_{cam}=5$ m, and FOV = 40 degrees. Also, the number of time steps for the initial data acquisition and model generation stage was $T_{init}=9000$ (equivalently, the duration of completion of 50 orbits by the chaser), which was determined by trial and error so that the generated GP models could provide reliable estimates. The data set $D_{r}$ had five dimensions (k,x,y,θ,r) and the maximum size was S=9000.

#### 3.2.2. Simulation Results

**Figure 7:**
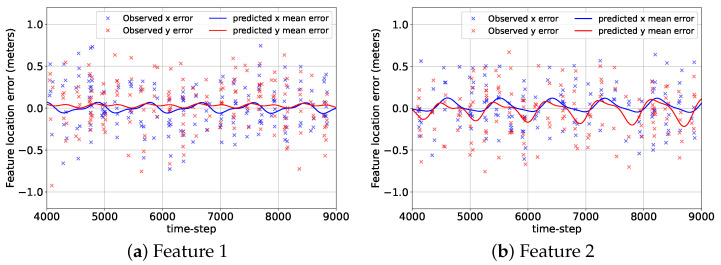

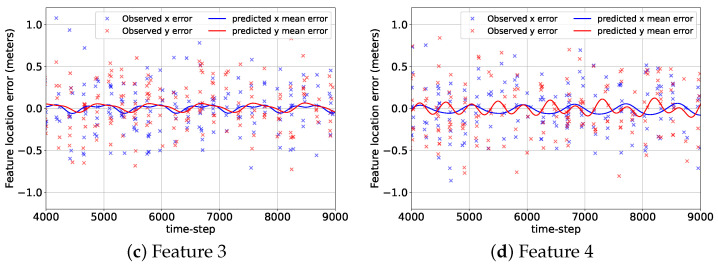
The errors associated with the predicted mean location components of the features from the GP-feature models and the measurement errors for the 2D case from k=4000 to k=9000.

Figure 7 shows the errors associated with the mean predicted location components of the features from their corresponding GP-feature models and the camera measurement errors at the beginning of the BO implementation stage, k=9001, for the 2D case. Although the GP-feature models provided the estimated feature locations for the entire duration of the mission (9000 + 225 time steps), the time span of the error plots shown in Figure 7 was chosen to be from k=4000 to k=9000 so that the reader can have a clear picture of the prediction performance of the GP-feature models. During the initial data acquisition and model generation stage from k=1 to k=9000, the camera detected features 1, 2, 3, and 4 only 300, 267, 322, and 285 times. Still, even with these small numbers of detection events, the GP-feature models could create very accurate feature location prediction models. Hence, in Figure 7, the prediction errors are close to zero, which indicate the effectiveness of utilizing a GP with the standard periodic kernel for generating feature location estimation models.

**Figure 8:**
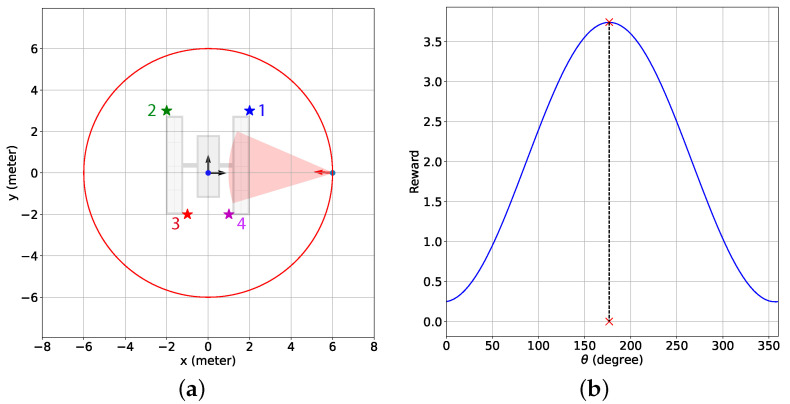

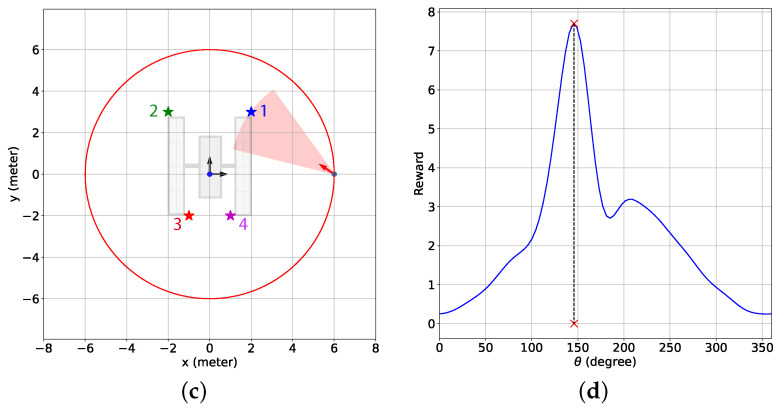
The simulation scenarios and the corresponding mean predicted rewards from the GP-feature models for the 2D case with the sinusoidal and the combined reward models at the beginning of the BO implementation stage (k=9001). The chaser is located at ${\left[6,0\right]}^{T}$ meters. (**a**) Simulation scenario with the sinusoidal reward; (**b**) the mean predicted reward from the GP-reward model with the sinusoidal reward; (**c**) simulation scenario with the combined reward; (**d**) the mean predicted reward from the GP-reward model with the combined reward. In (**a**,**c**), the red shaded regions indicate the camera coverage areas, and the red arrows represent the camera direction vectors. Also, the cross symbols on the x-axes in (**b**,**d**) represent the camera directional angles with the highest estimated rewards predicted by BO from the GP-feature models.

Next, we present the simulation scenarios at the beginning of the BO implementation stage (k=9001) and the associated GP-reward distribution over the camera directional angle in Figure 8 for the 2D case with the sinusoidal and the combined reward models. Figure 8a shows that for the sinusoidal reward, the camera is pointed in between features 1 and 4, which leads to no feature detection at this instance. The underlying reason is that although feature 1 is the closest feature to the chaser, features 1 and 4 have similar distances from the chaser, resulting in these features having a similar influence on the sinusoidal reward. Hence, the maximum predicted reward is attained by pointing the camera at neither feature 1 nor feature 4 but in the middle. From Figure 8b, it is observed that this camera directional angle is θ=176.64 degrees, denoted by the red cross symbol on the x-axis, which has the highest estimated reward predicted by BO from the GP-reward model. On the other hand, for the combined reward in Figure 8c,d, we notice that the camera is pointed toward feature 1 as the estimated highest reward predicted by BO from the GP-reward model is obtained for θ=146.25 degrees. Due to the inclusion of the feature detection reward, which was designed based on historical feature detection data, the chaser can determine that detecting feature 1 at this instance results in a better reward because feature 1 is the closest, and both features cannot be detected simultaneously. In Figure 8d, we can see another peak with a smaller reward on the right, and the corresponding θ represents the camera directional angle appropriate for detecting feature 4. Figure 8 helps to realize how BO with the GP-reward model can lead to determining the proper camera directional angles required for detecting the features.

**Figure 9:**
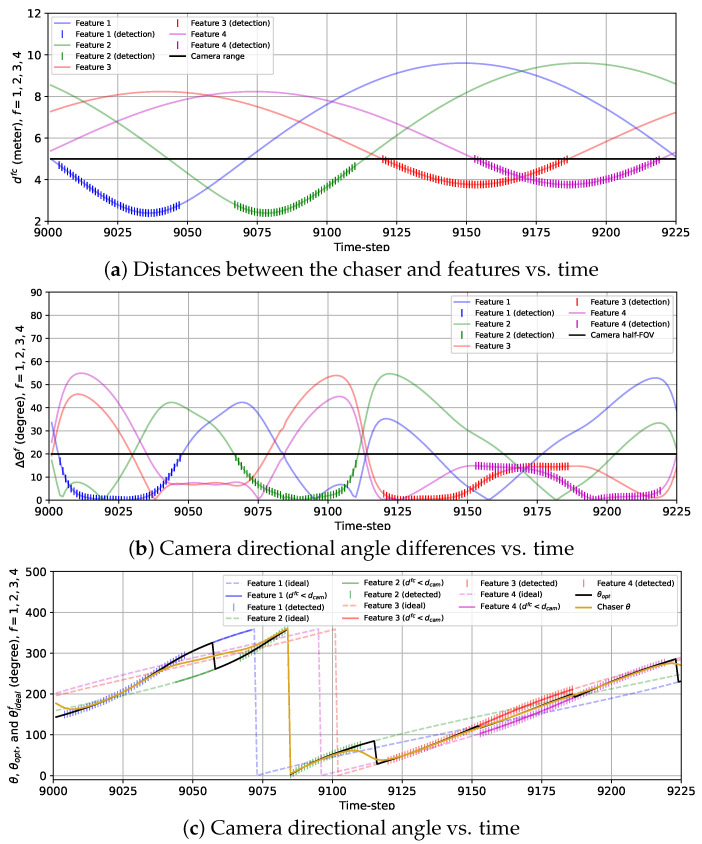
Plots showing the variations in (**a**) the distances between the chaser and features, (**b**) the camera directional angle differences, and (**c**) the camera directional angle for the 2D case with the sinusoidal reward from k=9001 to k=9225. (**c**) also provides the variations in the optimal camera directional angle and the ideal camera directional angles for all features.

In Figure 9, the variations in the actual distances between the chaser and the features ${\left\{d^{fc}\right\}}_{f=1}^{4}$, the true camera directional angle differences for all features ${\left\{\Delta \Theta^{f}\right\}}_{f=1}^{4}$, and the camera directional angle θ along with the true ideal camera direction angles for each feature, ${\left\{\theta_{ideal}^{f}\right\}}_{f=1}^{4}$ and the optimal camera directional angle $\theta_{opt}$ are presented for the 2D case with the sinusoidal reward model ($w_{det}=0$). Together, these three plots show that at the beginning of the BO implementation stage, both features 1 and 4 are almost at the same distance (less than $R_{feat}$) from the chaser. Hence, both $\Delta \Theta^{1}$ and $\Delta \Theta^{4}$ are quite high because these two features have a similar influence on $r_{sin}$, which we discussed in Section 2. As the chaser moves toward feature 1, the camera is gradually pointed toward this feature, and $\Delta \Theta^{1}$ drops starting from k=9001 to k=9011. From k=9012 to k=9036, $\Delta \Theta^{1}$ is close to zero and $\theta \approx \theta_{ideal}^{1}=\theta_{opt}$ because other features are more than an $R_{feat}$ distance away from the chaser. As the chaser moves along the trajectory, it gets away from feature 1 starting from k=9037 and travels toward feature 2. During this transition, the camera is gradually turned away from feature 1 to feature 2 as θ begins to change toward $\theta_{ideal}^{2}$ from $\theta_{ideal}^{3}$, which is indicated by increasing $\Delta \Theta^{1}$ and decreasing $\Delta \Theta^{2}$ from k=9037 until k=9083. During this transition, due to θ changing gradually instead of following $\theta_{opt}$, neither feature 1 nor 2 is detected from k=9048 to k=9066 since they cannot be detected simultaneously. From k=9084 to k=9101, only feature 2 has a significant influence on $r_{sin}$, so $\Delta \Theta^{2}$ is very small (<3 degrees), and $\theta \approx \theta_{ideal}^{2}=\theta_{opt}$. After that, $\Delta \Theta^{2}$ quickly grows, and at the same time, $\Delta \Theta^{3}$ drops because θ starts to move toward $\theta_{ideal}^{3}$ from $\theta_{ideal}^{2}$, which signifies that the camera is being turned toward feature 3 from feature 2 as the chaser moves toward feature 3. Similar to features 1 and 2, features 2 and 3 cannot be detected at the same time, and as a result, during this transition, no feature is detected from k=9111 to k=9119. We observe that from k=9121 to k=9147, $\Delta \Theta^{3}$ remains steady and very small ($\theta \approx \theta_{ideal}^{3}=\theta_{opt}$) but begins to rise as the chaser moves toward feature 4 after k=9147. Unlike features 1 and 2 or features 2 and 3, features 3 and 4 can be detected simultaneously, resulting in the detection of both features from k=9153 to k=9186. During this time interval, θ gradually shifts toward $\theta_{ideal}^{4}$ from $\theta_{ideal}^{3}$, and consequently, $\Delta \Theta^{3}$ rises, and $\Delta \Theta^{4}$ drops. Next, $\Delta \Theta^{4}$ drops further as feature 4 becomes the only feature within the $R_{feat}$ distance from the chaser ($\theta \approx \theta_{ideal}^{4}=\theta_{opt}$). At the end of the simulation, $\Delta \Theta^{4}$ increases, and $\Delta \Theta^{1}$ decreases due to the camera being turned toward feature 1 as the chaser moves closer to feature 1.

**Figure 10:**
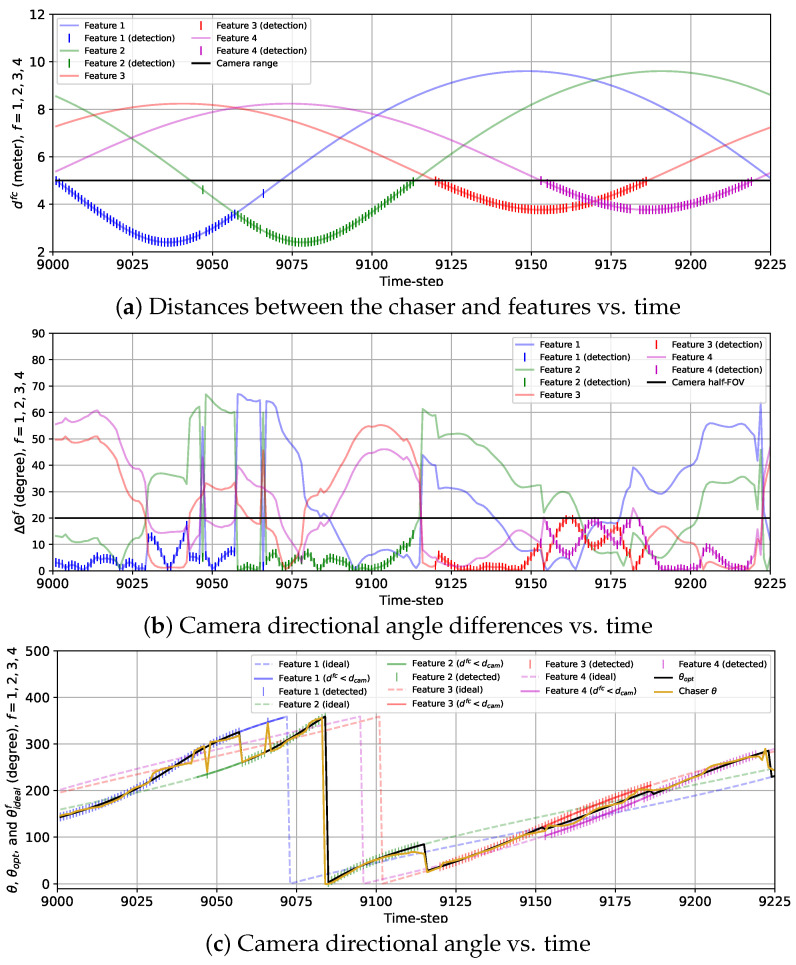
Plots showing the variations in (**a**) the distances between the chaser and features, (**b**) the camera direction angle differences, and (**c**) the camera directional angle for the 2D case with the combined reward from k=9001 to k=9225. (**c**) also provides the variations in the optimal camera directional angle and the ideal camera directional angles for all features.

Similar to Figure 9, we show the variations in ${\left\{d^{fc}\right\}}_{f=1}^{4}$, ${\left\{\Delta \Theta^{f}\right\}}_{f=1}^{4}$, and θ along with ${\left\{\theta_{ideal}^{f}\right\}}_{f=1}^{4}$ and $\theta_{opt}$ in Figure 10 for the 2D case with the combined reward model. The most noteworthy difference between the sinusoidal and combined reward models we notice from Figure 9 and Figure 10 is that with the combined reward model, feature 1 is detected from k=9048 to k=9057 (except at k=9047), and feature 2 is detected from k=9058 to k=9065, whereas the sinusoidal reward does not lead to any feature detection within this duration. We can also observe the instant rise in $\Delta \Theta^{1}$ and drop in $\Delta \Theta^{2}$ in Figure 10b and sudden change in θ from $\theta_{ideal}^{1}$ to $\theta_{ideal}^{2}$ in Figure 10c at k=9058, because the chaser points the camera from feature 1 to feature 2 at that instance. This change in θ matches with the change in $\theta_{opt}$ due to the combined reward model as seen from Figure 10c. We can also notice the sudden change in $\Delta \Theta^{2}$ and $\Delta \Theta^{3}$ for feature 2 and 3 happening at k=9116 from Figure 10b. At this instance, θ experiences a rapid change which coincides with the change in $\theta_{opt}$ as shown in Figure 10c. For features 3 and 4, the detection performance with the sinusoidal and the combined reward models are alike. Another contrast we observe between the sinusoidal and the combined rewards is that ${\left\{\Delta \Theta^{f}\right\}}_{f=1}^{4}$ obtained with the combined reward fluctuates a lot compared to the smooth change in ${\left\{\Delta \Theta^{f}\right\}}_{f=1}^{4}$ with time for the sinusoidal reward. This is expected due to the feature detection reward, which was designed based on historical feature detection information, as mentioned in Section 2. These fluctuations can also be observed in Figure 10c, which shows that θ follows $\theta_{ideal}^{f}$ for the closest feature with occasional small deviations. Additionally, unlike the previous case, some anomalies occur in this case. For example, at k=9046 and k=9066, features 2 and 1 are detected, respectively, when neither of them is the closest feature. Also, feature 3 is not detected at k=9162 because θ is too deviated from $\theta_{ideal}^{3}$. Similarly, the chaser fails to detect feature 4 at k=9154, k=9182, and k=9183 because θ diverged from $\theta_{ideal}^{4}$. These anomalies are attributed to the fluctuations in θ resulting from the feature detection reward. However, these occasional anomalies are not very concerning as they happened only in 6 of 225 time steps. These anomalies can be avoided by using a bigger training data set for the GP-reward model.

**Figure 11:**
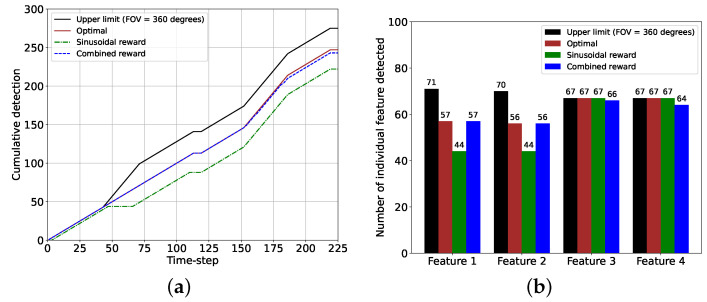
Performance comparisons between the sinusoidal and the combined reward models for the 2D case and for 225 time steps from k=9001 and k=9225. (**a**) Plot showing the cumulative feature detection for two reward models. (**b**) Histogram showing the number of detection events of each feature for two reward models.

The cumulative and individual feature detection performance comparisons between the sinusoidal and the combined rewards, along with the optimal performance and the corresponding upper limits, are provided in Figure 11. Here, we obtained the upper limits by setting the camera FOV = 360 degrees, meaning that in that case, a feature was considered detected if it was within the camera range $d_{cam}$. Moreover, the optimal performance indicated the feature detection performance if the chaser maintained the camera direction according to $\theta_{opt}$. From the cumulative feature detection performance plot in Figure 11a, we observe that the total numbers of feature detection events were 225 and 243 with the sinusoidal and the combined reward models, whereas the optimal performance value was 247 and the upper limit was 275. One can notice that the plot for the combined reward model almost overlaps the plot for the optimal case, and the difference between these two plots is due to the feature detection anomalies associated with the combined reward. Figure 11b shows that each of features 1 and 2 was detected 44 times with the sinusoidal reward whereas the combined reward detected these two features 13 and 12 more times, respectively, matching the performance of the optimal case. The upper limit was much higher for these two features because they could not be detected simultaneously with the camera FOV = 40 degrees. However, features 3 and 4 were detected 67 times by the sinusoidal reward models, which was the same as the optimal performance and the upper limit (the combined reward showed marginally inferior performance for these two features due to the anomalies). The detection performance for these two features with the reward models was very similar to the optimal case and the upper limit because the positions of these features, the camera FOV, and the range allowed these features to be detected together, and the proposed algorithm managed to determine the correct camera directional angles. The performance comparisons showed that the combined reward model was superior to the sinusoidal reward models (feature detection improved by as much as (57 − 44)/44 = 30%), given that a considerably large data set was provided.

### 3.3. Three-Dimensional Case

#### 3.3.1. Simulation Setup

For the 3D case, the initial feature positions were given as follows: $p^{1}\left(1\right)={\left[2,-6,-4\right]}^{T}$ meters, $p^{2}\left(1\right)={\left[-2,-6,4\right]}^{T}$ meters, $p^{3}\left(1\right)={\left[-2,4,2\right]}^{T}$ meters, and $p^{4}\left(1\right)={\left[2,4,-2\right]}^{T}$ meters. The unit vector of the rotation axis of the features was ${\left[\cos\left(20^{o}\right),0,\sin\left(20^{o}\right)\right]}^{T}={\left[0.3420,0,0.9397\right]}^{T}$ meters, and the features rotated with respect to the rotation axis in the clockwise direction. The radius of the relative chaser orbit was 12 m and the parameters in Equations (1)–(3) which define the chaser trajectory were $A_{0}=6$ m, $B_{0}=6\sqrt{3}$ m, α=0, β=−90 degrees, and n=0.05 degree/s. The choices of values of $A_{0}$, $B_{0}$, α, and β to create a circular relative chaser orbit are discussed in detail in Appendix B. The chaser performed a rotation along the predefined trajectory in the clockwise direction viewed from the top. Also, the camera range was $d_{cam}=10$ meters and FOV = 50 degrees. The duration of the initial data acquisition and model generation stage $T_{init}$ for the sinusoidal reward was 9000 time steps (50 chaser orbits), whereas $T_{init}$ = 16,200 (90 chaser orbits) for the combined reward model. The maximum size *S* of the data set $D_{r}$ was also 9000 and 16,200 for the sinusoidal and the combined rewards. Similar to the 2D case, the $T_{init}$ values for the 3D cases were determined by trial and error. We considered a more significant value for the combined reward because with a smaller $D_{r}$, the combined reward did not perform better than the sinusoidal reward. It was apparent that the combined reward could perform better than the sinusoidal reward but to do so, it required more data than the sinusoidal reward.

#### 3.3.2. Simulation Results

**Figure 12:**
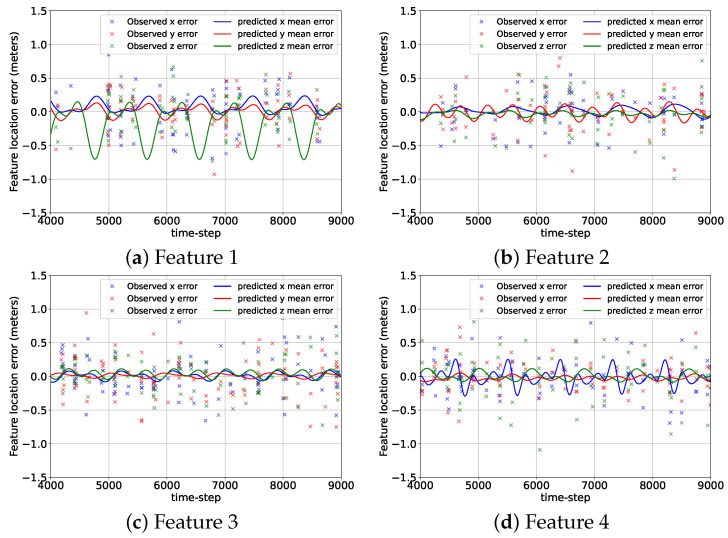
The errors associated with the predicted mean location components of the features from the GP-feature models and the measurement errors for the 3D case from k=4000 to k=9000.

In Figure 12, we provide the errors of the three mean predicted components of the feature locations from their corresponding GP-feature models and the observation errors after 9000 time steps of random data collection for the 3D case. Similar to Figure 7, the time span of the plots in Figure 12 is from k=4000 to k=9000. For the 3D case, from k=1 to k=9000, the camera detected features 1, 2, 3, and 4 only 160, 148, 208, and 179 times. Still, the GP-feature prediction models generated from these observations were pretty accurate for all the features and all the components. The only exception was the prediction model for the z component of feature 1, where the GP-feature model had a considerable difference from the truth due to the lack of measurements. Figure 12 demonstrates that similar to the 2D case, GP can be utilized for the 3D case to create feature location estimation models, which have minimal differences from the truth for most parts.

**Figure 13:**
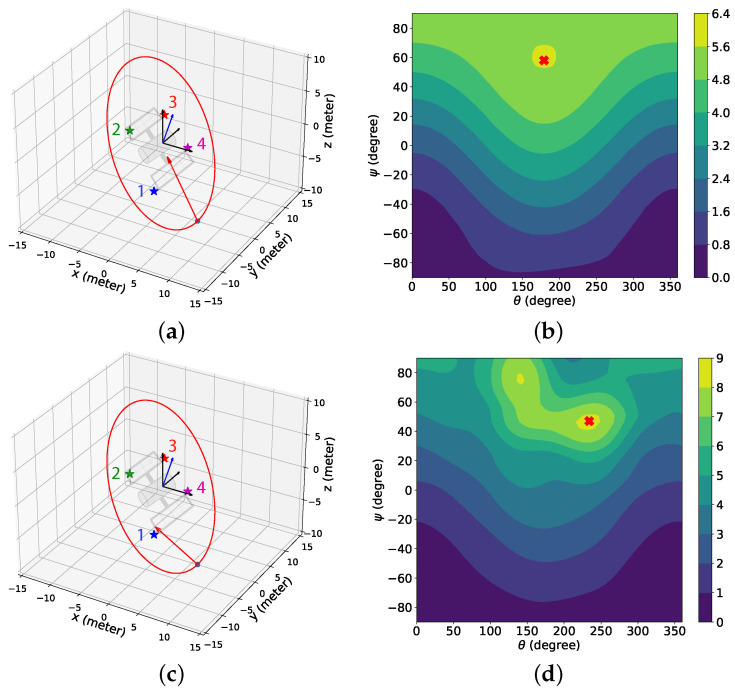
The simulation scenarios and the corresponding mean predicted rewards from the GP-feature models for the 3D case with the sinusoidal and the combined reward models at the beginning of the BO implementation stage (k=9001 for the sinusoidal reward, *k* = 16,201 for the combined reward). The chaser is located at ${\left[6,0,-10.39\right]}^{T}$ meters. (**a**) Simulation scenario with the sinusoidal reward. (**b**) The mean predicted reward from the GP-reward model with the sinusoidal reward. (**c**) Simulation scenario with the combined reward. (**d**) The mean predicted reward from the GP-reward model with the combined reward. The cross symbols in (**b**,**d**) represent the camera directional angles with the highest estimated rewards predicted by BO from the GP-feature models.

Figure 13 provides the simulation scenarios at the beginning of the BO implementation stage for the 3D case with the sinusoidal (k=9001) and the combined (*k* = 16,201) reward models, and the contour plot of the associated GP-reward distribution over the camera directional angles. Due to the resemblance between the simulation setup of the 2D and 3D cases, the results provided in Figure 13 are similar to Figure 8. Figure 13a illustrating the simulation scenario for the sinusoidal reward shows that the camera is pointed between features 1 and 4. According to Figure 13b, the corresponding camera directional angle pair {θ,ψ}={178.96,64.96} degrees has the highest estimated reward (denoted by the red cross symbol) predicted by BO from the GP-reward model because these features are almost equidistant from the chaser and therefore have a similar influence on the sinusoidal reward. The chaser tries to obtain the highest reward by pointing the camera between feature 1 and feature 4, although none of the two features are detected. On the contrary, for the combined reward in Figure 13c,d, it is observed that the camera is pointed toward feature 1 as the estimated highest reward predicted by BO from the GP-reward model is obtained for {θ,ψ}={234.06,47.30} degrees which leads to the detection of feature 1. We also see from Figure 13d that there exists another peak with a smaller reward on the top left, which indicates the camera directional angles for detecting feature 4.

**Figure 14:**
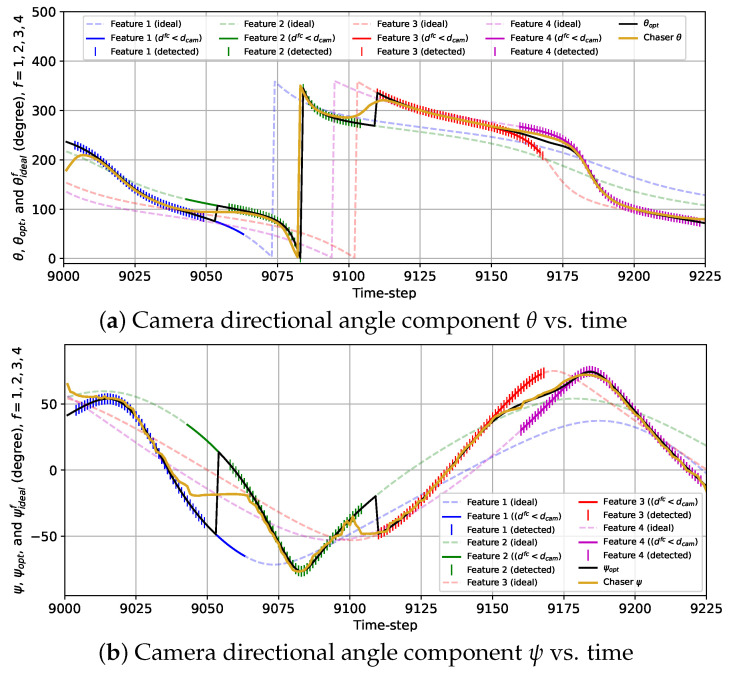
Variations in the camera directional angle components with time for the 3D case with the sinusoidal reward model from k=9001 to k=9225.

The variations in the camera directional angles θ and ψ, as well as the optimal and the ideal camera directional angles for all features vs. time for the 3D case with the sinusoidal reward are provided in Figure 14. The trends of the variations in θ and ψ are quite similar to that of θ for the 2D case with the sinusoidal reward. Initially, θ and ψ move toward $\theta_{ideal}^{1}=\theta_{opt}$ and $\psi_{ideal}^{1}=\psi_{ideal}$ as the chaser moves toward feature 1. From k=9009 to k=9040, $\theta \approx \theta_{ideal}^{1}=\theta_{opt}$ and $\psi \approx \psi_{ideal}^{1}=\psi_{opt}$ as the rest of the features are located further than the distance $R_{feat}$ from the chaser. From k=9041 to k=9066, the camera is turned away from feature 1 toward feature 2, and during this change in the camera directional angles, the camera cannot detect either of the features from k=9050 to k=9057. Next, feature 2 is tracked from k=9067 to k=9100 ($\theta \approx \theta_{ideal}^{2}=\theta_{opt}$ and $\psi \approx \psi_{ideal}^{2}=\psi_{opt}$). Then, the camera is slowly turned toward feature 3, and from k=9105 to k=9109, the chaser fails to detect features 2 and 3. Afterward, $\theta \approx \theta_{ideal}^{3}=\theta_{opt}$ as well as $\psi \approx \psi_{ideal}^{3}=\psi_{opt}$ are maintained from k=9113 till k=9154, followed by the camera being gradually turned toward feature 4. During this transition, features 3 and 4 are simultaneously detected. Finally, the camera is kept pointed toward feature 4 from k=9175 until the end of the simulation ($\theta \approx \theta_{ideal}^{4}=\theta_{opt}$ and $\psi \approx \psi_{ideal}^{4}=\psi_{opt}$).

**Figure 15:**
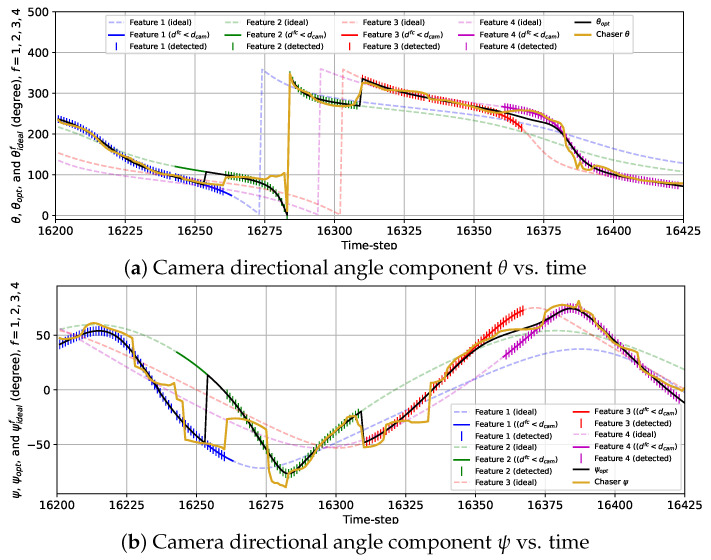
Variations in the camera directional angle components with time for the 3D case with the combined reward model from *k* = 16,201 to *k* = 16,425.

The plots representing the variations in θ and ψ with the combined reward model in Figure 15 show the frequent fluctuations in θ and ψ from the ideal camera directional angles. Due to these fluctuations, we observe some anomalies regarding the detection of the features. For example, the chaser fails to detect feature 1 at *k* = 16,242 due to ψ being too deviated from $\psi_{ideal}^{1}=\psi_{opt}$. Also, the camera is expected to turn toward feature 2 from feature 1 at *k* = 16,254 following $\theta_{opt}$ and $\psi_{opt}$ as feature 2 is closer to the chaser than feature 1 starting from that instance. However, the chaser lags for seven time steps in realizing this transition and starts detecting feature 2 from *k* = 16,261. Additionally, feature 3 is not detected at *k* = 16,333 due to ψ being diverged from $\psi_{ideal}^{3}=\psi_{opt}$. Except for these anomalies, the overall detection performance observed from this simulation is quite satisfactory.

**Figure 16:**
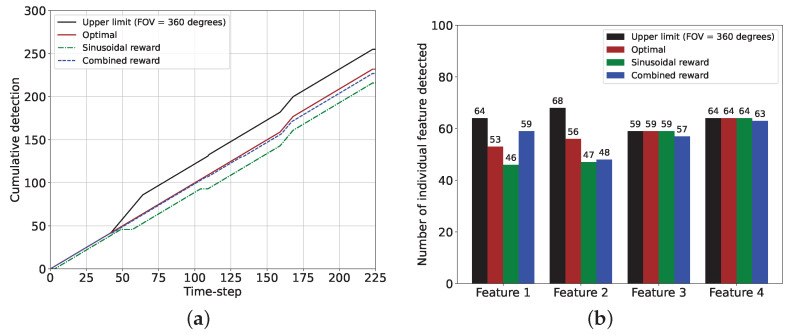
Performance comparisons between the sinusoidal and the combined reward models for the 3D case and for 225 time steps (sinusoidal: *k* = 9001 to *k* = 9225, combined: *k* = 16,201 to *k* = 16,425). (**a**) Plot showing the cumulative feature detection events for two reward models. (**b**) Histogram showing the number of detection events of each feature for two reward models.

The cumulative and individual feature detection performance for the sinusoidal and the combined rewards with the optimal performance and the corresponding upper limits are provided in Figure 16. The cumulative feature detection performance in Figure 16a shows that the performance of the combined reward is very close to the optimal performance whereas the sinusoidal reward offers inferior performance due to the model’s failure in detecting feature during the gradual turning of the camera from features 1 to 2 and feature 2 to 3. Hence, Figure 16b shows that with the combined reward, features 1 and 2 are detected more frequently than the sinusoidal reward. For features 3 and 4, the detection performance values of the two reward models are comparable, which also match the optimal performance. Therefore, it is evident that the combined reward performs better (as much as (59 − 46)/46 = 28%) than the sinusoidal reward. Still, the combined reward model requires a bigger data set to perform better than the sinusoidal reward.

## 4. Conclusions

This paper presented a Bayesian Optimization (BO)-based framework called the Space Object Chaser-Resident Assessment Feature Tracking (SOCRAFT) algorithm for detecting features of a non-cooperative and rotating space object from a chaser spacecraft in a predefined orbit carrying a monocular camera and a single-beam LIDAR. Rewards were assigned to the chaser states to obtain the camera directional angles. In particular, the reward model was designed based on two reward components: the feature detection and the sinusoidal rewards. The feature detection reward was assigned based on historical feature detection information, and the sinusoidal reward was calculated from the difference between the actual and estimated ideal chaser states. Gaussian Process (GP) models were designed to predict the required estimated feature locations to obtain the estimated ideal chaser state. The target rotational period was estimated from the Fast Fourier Transform algorithm. Also, the reward distribution model over the chaser state, called the GP-reward model, was created, which was utilized by the BO to determine the camera directional angles.

To demonstrate the efficacy of the proposed (SOCRAFT) algorithm, simulations were conducted for the 2D and 3D cases with the sinusoidal and the combined reward models. Due to similar simulation setups, simulation results acquired from the two cases were comparable. Overall, the obtained results demonstrated that the SOCRAFT algorithm could detect the maximum number of features within the limited FOV and range. Moreover, the results obtained with the sinusoidal reward showed that the camera directional angle changed gradually and could reach very close to the ideal camera directional angles for a feature if the other features were further than the radius of influence from the chaser. The gradual change in the camera directional angles with the sinusoidal reward also indicated that when the chaser moved from one feature toward another, the chaser may fail to detect both features if the features were not simultaneously detectable. On the contrary, the combined reward model caused the camera direction angles to fluctuate more frequently, and occasional anomalies were also observed. Still, this reward model could detect the features more successfully and the number of feature detection events with the combined reward model was as much as 30% higher than that of the sinusoidal reward model. However, a bigger training data set was required for the combined reward to achieve this superiority over the sinusoidal reward.

As a pioneer work on utilizing a combination of Gaussian Process and Bayesian Optimization for feature detection, this paper considered a relatively simple case to demonstrate the fundamental concept of the proposed algorithm. In the future, we will gradually remove the assumptions made in this study. In particular, we plan to implement suitable image processing algorithms and extend our work to a varying number of features. Furthermore, we will design optimal relative trajectories for the chaser that consider both time and energy for the mission tasks.

## Figures and Tables

**Table 1:** The format of the data set $D_{cam}$ for storing the camera measurement information.

Time Step	Noisy Feature Location	Feature Index
1	${\tilde{p}}^{f}\left(1\right)$	f(1)
2	${\tilde{p}}^{f}\left(2\right)$	f(2)
⋮	⋮	⋮
*k*	${\tilde{p}}^{f}\left(k\right)$	f(k)

**Table 2:** The format of the training data set $D_{r}$ for the GP-reward model Λ.

Input Training Data (Chaser States)						Output Training Data
Time Step	Chaser Location Components			Camera Dir.		Reward r
	x Component	y Component	z Component	θ	ψ	
1	x(1)	y(1)	z(1)	θ(1)	ψ(1)	r(1)
2	x(2)	y(2)	z(2)	θ(2)	ψ(2)	r(2)
⋮	⋮	⋮	⋮	⋮	⋮	⋮
*k*	x(k)	y(k)	z(k)	θ(k)	ψ(k)	r(k)

## Data Availability

The data sets presented in this article are not readily available because the data are part of an ongoing study. Requests to access the data sets should be directed to xiaoli.bai@rutgers.edu.

## Abbreviations

The following abbreviations are used in this manuscript:

BO	Bayesian Optimization
GP	Gaussian Process
FFT	Fast Fourier Transform
SOCRAFT	Space Object Chaser-Resident Assessment Feature Tracking
FOV	Field of view
LIDAR	Light Detection and Ranging
LEO	Low Earth Orbit
RSO	Resident Space Object
NASA	National Aeronautics and Space Administration
ISAM	In-Space Servicing, Assembly, and Manufacturing
PE	Pursuit–Evasion
SDA	Space Domain Awareness
SLAM	Simultaneous Localization and Mapping
RANSAC	Random Sample Consensus
SIFT	Scale Invariant Feature Transform
ISS	International Space Station
ASLAM	Active Simultaneous Localization and Mapping
MPC	Model Predictive Control
SBMPO	Sampling-Based Model Predictive Optimization
EKF	Extended Kalman Filter
UKF	Unscented Kalman Filter
ML	Machine Learning
RL	Reinforcement Learning
**Nomenclature**	
H	Target Hill frame
$\omega_{f}$, *P*	Angular velocity and rotational period of the target, respectively
$p^{c}$, $p^{f}$	Positions of the chaser and feature *f* in H
$p^{fc}$	Relative position of feature *f* with respect to the chaser in H
$p_{x}^{fc},p_{y}^{fc},p_{z}^{fc}$	Spatial components of $p^{fc}$
*k*	Time step index
$t_{k}$	Time instance at time step *k*
$T_{init}$, *T*	Total time steps for the initial data collection and the complete duration of the simulation, respectively
$A_{0}$, $B_{0}$, α, β, $y_{off}$	Integral constants associated with the Clohessy–Wiltshire equations
*x*, *y*, *z*	Spatial components of $p^{c}$
θ, ϕ	Camera directional angle components
$\theta_{opt}$, $\phi_{opt}$	Optimal camera directional angle components
$\theta_{ideal}^{f}$, $\phi_{ideal}^{f}$	Ideal camera directional angle components for feature *f* given that the camera direction vector is exactly pointed toward feature *f*
$u_{cam}$	Unit vector in the direction of the camera orientation in H
$X^{c}$	Chaser state
$d^{fc}$	Distance between the chaser and feature *f*
$d_{min}^{fc}$	Minimum distance between the chaser and feature *f*
$d_{cam}$	Camera range
FOV	Camera field of view
$\Delta \Theta^{f}$	Angle between $u_{cam}$ and $p^{fc}$
w	White Gaussian measurement noise
$\sigma_{m}$	Standard deviation of the measurement noise
Y	Output from the measurement model
$D_{chs}$, $D_{cam}$	Data sets for storing X and Y, respectively
$D_{r}$	Data sets for storing the chaser states and the associated rewards
*S*	Maximum size of $D_{r}$
*M*, $M_{det}$	Number of total and detected features, respectively
$r_{det}$, $r_{sin}$, *r*	Detection, total sinusoidal, and combined rewards, respectively
$r_{sin}^{f}$	Sinusoidal reward assigned to $X^{c}$ due to feature *f*
$r_{max}$	Constant maximum reward assigned for each detected feature
$w_{det}$	Constant weight for adjusting the priority of the detection reward
$w_{d}^{f}$	Distance weight associated with feature *f*
$R_{feat}$	Radius of influence
$\Gamma_{i}^{f}$	Gaussian Process model for predicting the *i*th position component of feature *f*
Λ	Gaussian Process model for estimating the reward distribution over X
$K_{f}$, *K*	Kernel functions associated with $\Gamma_{i}^{f}$ and Λ, respectively
$\sigma_{k}$, $\sigma_{p^{c}},\sigma_{\theta },\sigma_{\phi }$	Smoothness hyper-parameters in the kernel functions
$l_{k}$, $l_{p^{c}},l_{\theta },l_{\phi }$	Length-scale hyper-parameters in the kernel functions
$F_{max}$	Frequency with the highest magnitude from the FFT analysis
$a^{*}$	Optimal action according to BO
A	Action space
α(X,κ)	The Upper Confidence Bound acquisition function
μ(X), σ(X)	Mean and standard deviation of *r* from Λ
$\hat{•}$	Estimated value
$\tilde{•}$	Measured value

## Appendix A. Derivation of the Expression of the Reverse Sigmoid Function $w_{d}^{f}$ in Equation (18)

Given that

$$w_{d}^{f}=\left\{\begin{array}{cc} 0.01, & \mathrm{if}\;{\hat{d}}^{fc}=R_{feat}\\ 0.99, & \mathrm{if}\;{\hat{d}}^{fc}={\hat{d}}_{min}^{fc}\\ 0.5, & \mathrm{if}\;{\hat{d}}^{fc}=\frac{R_{feat}+{\hat{d}}_{min}^{fc}}{2} \end{array}\right.$$

find the expression of the reverse sigmoid function $w_{d}^{f}$.

**Proof.** The general expression of a reverse sigmoid function $w_{d}^{f}$ can be written as follows:

(A1) $$w_{d}^{f}={\left[1+\exp\left[a({\hat{d}}^{fc}-b)\right]\right]}^{-1}$$

where *a* and *b* are constant parameters defining a specific reverse sigmoid function. If b=0, then $w_{d}^{f}=0.5$ at ${\hat{d}}^{fc}=b=0$. Hence, to satisfy the condition $w_{d}^{f}=0.5$ at ${\hat{d}}^{fc}=\frac{R_{feat}+{\hat{d}}_{min}^{fc}}{2}$, we can set $b=\frac{R_{feat}+{\hat{d}}_{min}^{fc}}{2}$. Next, to obtain the value of *a*, we use the the first condition, that is $w_{d}^{f}=0.01$ if ${\hat{d}}^{fc}=R_{feat}$,

$$\begin{array}{cc} \; & {\left[1+\exp\left[a(R_{feat}-b)\right]\right]}^{-1}=0.01\\ \Rightarrow \; & 1+\exp\left[a(R_{feat}-b)\right]=\frac{1}{0.01}\\ \Rightarrow \; & \exp\left[a(R_{feat}-b)\right]=\frac{1}{0.01}-1\\ \Rightarrow \; & a\left(R_{feat}-b\right)=\ln\left(\frac{0.99}{0.01}\right)=\ln\left(99\right)\\ \Rightarrow \; & a=\frac{\ln(99)}{R_{feat}-b}=\frac{\ln(99)}{R_{feat}-\frac{R_{feat}+{\hat{d}}_{min}^{fc}}{2}}=\frac{2\ln(99)}{R_{feat}-{\hat{d}}_{min}^{fc}} \end{array}$$

By replacing *a* and *b* with their corresponding expressions in Equation (A1), we can write the following expression of $w_{d}^{f}$:

(A2) $$w_{d}^{f}\left(k\right)={\left[1+\exp\left(\frac{2\ln(99)}{R_{feat}-{\hat{d}}_{min}^{fc}}\left({\hat{d}}^{fc}\left(k\right)-\frac{R_{feat}+{\hat{d}}_{min}^{fc}}{2}\right)\right)\right]}^{-1}$$

□

## Appendix B. Derivation of the Necessary Conditions for Creating a Natural Closed Relative Chaser Orbit of Circular Shape

If we want to create a 3D circular natural closed relative chaser orbit centered at the origin of the target Hill frame H with radius *R*, we need to consider the following:

- $y_{off}=0$ in Equation (2).
- x(t), y(t) and y(t) satisfies the constraint $x{\left(t\right)}^{2}+y{\left(t\right)}^{2}+z{\left(t\right)}^{2}=R^{2}$.

Putting the expressions of x(t), y(t), and z(t) from (1)–(3) in $x{\left(t\right)}^{2}+y{\left(t\right)}^{2}+z{\left(t\right)}^{2}=R^{2}$, we obtain:

$$\begin{array}{cc} \; & A_{0}^{2}\cos^{2}\left(nt+\alpha \right)+4A_{0}^{2}\sin^{2}\left(nt+\alpha \right)+B_{0}^{2}\cos^{2}\left(nt+\beta \right)=R^{2}\\ \Rightarrow \; & A_{0}^{2}\left(\cos^{2}\left(nt+\alpha \right)+\sin^{2}\left(nt+\alpha \right)\right)+3A_{0}^{2}\sin^{2}\left(nt+\alpha \right)+B_{0}^{2}\cos^{2}\left(nt+\beta \right)=R^{2}\\ \Rightarrow \; & A_{0}^{2}+3A_{0}^{2}\sin^{2}\left(nt+\alpha \right)+B_{0}^{2}\cos^{2}\left(nt+\beta \right)=R^{2} \end{array}$$

Assuming that α=β or α=β+180 degrees and $3A_{0}^{2}=B_{0}^{2}$ (or $B_{0}=\pm \sqrt{3}A_{0}$), we can write:

$$\begin{array}{cc} \; & A_{0}^{2}+B_{0}^{2}=R^{2}\\ \Rightarrow & A_{0}^{2}+3A_{0}^{2}=R^{2}\\ \Rightarrow & 4A_{0}^{2}=R^{2}\\ \Rightarrow & A_{0}=\pm \frac{1}{2}R \end{array}$$

Therefore, in summary, for creating a 3D circular relative chaser orbit centered at the origin of the H frame satisfying the CW equations, we need the following conditions:

- $y_{off}=0$
- α=β or α=β+180 degrees
- $A_{0}=\pm \frac{1}{2}R$ and $B_{0}=\pm \sqrt{3}A_{0}$

Note that there might be other situations where the relative orbit is circular in 3D space.
